# Supplementary material for: Design and implementation of aerobic and ambient CO2-reduction as an entry-point for enhanced carbon fixation
Source: Nat Commun. 2025 Apr 1;16:3134. doi: 10.1038/s41467-025-57549-4 (PMC11961710; doi:10.1038/s41467-025-57549-4)
Supplement: Supplementary file 1 — Supplementary Information [file 41467_2025_57549_MOESM1_ESM.pdf]

Design and implementation of aerobic and ambient CO_2-reduction as an entry-point for enhanced carbon fixation

Satanowski and Marchal *et al.*

Supplementary Note 1. Initial *in vitro* prototyping of other CO_2-reduction pathways

Reductive formamide pathway

The reductive formamide pathway is a linear pathway and the simplest identified route for CO_2 reduction (Supplementary Fig. 2). Activation of bicarbonate by carbamoyl-phosphate synthetase is followed by the reduction to formamide and deamination to formate by formamidase. The new-to-nature reduction of carbamoyl-phosphate occurs either in one step or in two steps via carbamoyl-CoA as intermediate. Ammonia is used as the catalytic "carrier" of CO_2 for its reduction and release as formate. A major advantage of this pathway is its minimal overlap/interference with central carbon metabolism and the small number of required reaction steps (3-4 enzymes), while the engineering of the required phosphorylating formamide dehydrogenase (reaction A in Supplementary Fig. 2) from natural aldehyde dehydrogenases is expected to be quite challenging.

For the one-step reduction reaction (reaction A in Supplementary Fig. 2), we tested the ability of four enzyme candidates to accept carbamoyl-phosphate: Glyceraldehyde-3-phosphate dehydrogenase (GapA, Uniprot P0A9B2), gamma-glutamyl-phosphate reductase (ProA, Uniprot P07004) and N-acetyl-gamma-glutamyl-phosphate reductase (ArgC, Uniprot P11446) from *E. coli* did not show detectable activity in a spectrophotometric assay while *E. coli* aspartate-semialdehyde dehydrogenase (ASD, Uniprot P0A9Q9) showed a low basal activity of 3.4 nmol min^{-1} mg^{-1}. However, it was undetectable upon coupling to the subsequent formamidase reaction. For the two-step reduction via carbamoyl-CoA (reactions B1/B2 in Supplementary Fig. 2), we tested phosphate transacetylase (PTA, Uniprot P0A9M8) and phosphate propanoyltransferase (PduL, Uniprot Q21A54) for reaction B1 and propionaldehyde dehydrogenase (PduP, Uniprot Q21A49), but none of the enzymes was active on carbamoyl-phosphate. Therefore, we considered this pathway to be less feasible than the BKACE-based CORE cycle and focused on that pathway candidate instead. Overall, the BKACE cycle constituted the most promising pathway for CO_2 reduction in terms of feasibility, resource requirements, cofactor requirements, number of new-to-nature reactions and number of involved enzymes. Thus, we focused on this pathway design and that are able to catalyze the desired conversion of malonate semialdehyde and acetyl-CoA to the products formyl-CoA and acetoacetate (main text, Fig. 1e,f).

Oxaloacetate hydrolase cycle

The OAH cycle constitutes another promising pathway as it only needs one new-to-nature enzyme activity (Supplementary Fig. 3). It shares the same reactions from acetyl-CoA to malonate semialdehyde (MSA) as the BKACE cycle, but MSA is subsequently hydrolyzed into formate and acetate that in turn is reactivated to acetyl-CoA to close the cycle. With a total of 4-5 reactions, the cycle is comparably small and the irreversible cleavage of malonate semialdehyde provides a high driving force. Yet, engineering the new-to-nature activity might be challenging as the natural and the desired substrates are structurally more divers.

We performed experiments to check for the necessary promiscuity of oxaloacetate hydrolase to accept malonate. We tested oxaloacetate hydrolase from *Botrytis cinerea* (Uniprot Q6PNM8) [1], oxaloacetate hydrolase from *Cryphonectria parasitica* (Uniprot D5LIR7) [2] and a promiscuous 2,3-dimethylmalate lyase from *Aspergillus niger* (Uniprot Q2L887), which also accepts oxaloacetate as substrate [3]. We were unable to detect any activity with the novel substrate in spectrophotometric measurements. For all three candidates, there are structures available showing that active site residues form hydrogen bonds with both carboxyl groups of oxaloacetate. However, MSA bears a hydrogen atom instead of the second carboxyl group which probably impedes proper binding and coordination of the non-natural substrate. It might even lead to non-productive binding in the opposite direction due to the remaining carboxyl group. Additionally, the postulated mechanism with oxaloacetate contains an oxanion intermediate with the two negative charges of the oxanion and the carboxyl group being stabilized by Mn^{2+}. Due to the lack of the second carboxyl group, the second negative charge is missing, and the intermediate is much less stable. Therefore, we assessed this pathway to be non-feasible without bigger engineering efforts.

Aspartate-formate-lyase cycle

Another pathway design, named the AFL cycle, is also particularly promising (Supplementary Fig. 4): It requires equally few enzyme reactions (4 steps) and, importantly, an engineered enzyme has been previously reported to catalyze the key new-to-nature reaction required for release of formate in this pathway [4]. In contrast to the reductive formamide pathway, the AFL cycle has significant overlap with central metabolism, namely at the PEP/pyruvate-oxaloacetate node. The ATP-cost of the AFL cycle can be 66 % lower than that of the reductive formamide pathway, especially in microbes where ammonia re-assimilation can proceed via glutamate dehydrogenase, i.e. without ATP-investment.

Since the aspartate-formate-lyase reaction has already been engineered earlier from a tyrosine-phenol lyase by introducing two mutations [4], we re-cloned this mutated variant with an N- and C-terminal His-tag, overexpressed and purified it. Contrary to the literature report, we could not detect any activity, neither with aspartate, nor with its native substrate tyrosine. Addition of 0.1 mM PLP to the expression culture did not improve the outcome. Therefore, we assessed this pathway to be non-feasible without additional engineering efforts.

Methods for initial *in vitro* prototyping of other CO_2-reduction pathways

Spectrophotometric measurements for aspartate formate lyase (AFL): AFL activity was measured in a coupled-enzyme assay with lactate dehydrogenase (LDH). Reactions contained 50 mM $K_xH_yPO_4$ pH 8.0, 12 U LDH, 0.3 mM NADH, 0.05 mM pyrudoxal-5'-phosphate (PLP), 5 mM β-mercaptoethanol, 200 mM aspartate or 2 mM tyrosine and 0.3 – 1 mg/mL AFL. NADH depletion at 340 nm was observed in a CARY 60-UV-Vis Spectrophotometer and evaluated via the CARY kinetic program linear fit function.

Spectrophotometric measurements for oxaloacetate hydrolase (OAH): OAH activity was measured in a coupled-enzyme assay with formate dehydrogenase (FDH). MSA was produced in situ with β-alanine:pyruvate

transaminase (BPT). 100 mM MOPS/KOH pH 7.8, 5 mM pyruvate, 5 mM β-alanine, 1 mM NAD+, 0.05 mg/mL FDH and 0.2 – 0.9 mg/mL OAH were mixed in a cuvette and incubated for 2 min at 37 °C. The reaction was started with 0.05 mg/mL BPT and absorbance was measured over time at λ = 340 nm.

Spectrophotometric measurements for direct carbamoyl-phosphate reduction: Reactions contained 100 mM MOPS-KOH pH 7.0, 250 µM NAD(P)H, 50 mM carbamoyl phosphate and 10 or 20 µM enzyme. NAD(P)H depletion at 340 nm was observed in a CARY 60 UV-Vis Spectrophotometer and evaluated via the CARY kinetic program linear fit function.

Spectrophotometric analysis of carbamoyl-CoA production and depletion: Reactions contained 100 mM MOPS-KOH pH 7.0, 250 µM NADH, 1 mM CoA, 5 µM PduP, 1 µM PTA or PduL and 10 mM carbamoyl phosphate. NADH depletion at 340 nm was observed in a CARY 60 UV-Vis Spectrophotometer and evaluated via the CARY kinetic program linear fit function.

Berthelot assay for the detection of NH_3 in the formamidase reaction: Reactions contained 100 mM MOPS-KOH pH 7.0, 1 mM NADPH, 10 mM carbamoyl phosphate or formamide, 5 formamidase and 20 µM ASD. Timepoints were taken at 5, 30, 60 and 120 min by quenching 5 µL aliquots in 5 µL 0.4 M sodium phenolate and 5 µL 0.02 % w/v sodium nitroprusside. After all time points were taken, 5 µL of 0.5 % NaOCl were added. The reaction was incubated for 10 min at 42 °C, cooled to room temperature and absorbance at 640 nm was measured. For quantification, an ammonia dilution series was prepared in 100 mM MOPS pH 7.0 and treated in the identical fashion. After quantification, a linear fit was applied to the data from 5-60 min.

Supplementary Note 2. Revisiting the reaction mechanism of BKACE15

All BKACEs are part of the DUF849 Pfam family that has been classified into seven structural groups based on the active site composition [5]. BKACE15 belongs to structural group G1 that was reported to predominantly accept β-keto acids with apolar and noncharged terminal moieties, matching our observation of its activity on MSA. In an earlier BKACE study, the reaction mechanism of BKACE has been described based on 3-keto-5-aminohexanoate (KAH) cleavage enzyme (Kce) from *Candidatus Cloacimonas acidaminovorans* [6]. This enzyme belongs to structural group G2 that is characterized by having a very narrow substrate spectrum with substrates closely resembling KAH [5]. The first published structure from that study contained the native substrate KAH and Zn^{2+}, but no bound CoA or CoA ester (PDB 2Y7F). A second structure contained acetoacetate instead of KAH (PDB 2Y7G), however, also lacked the CoA ester co-substrate. In both structures, KAH and acetoacetate were coordinated on the Zn^{2+} in two different orientations (flipped about 90°). In contrast, in our structures, we only observed one orientation, where the carboxyl groups of acetoacetate or malonate were stabilized by Zn^{2+}, T85, S110 and N112 (corresponding to S82, T106 and N108 in Kce, see Supplementary Fig. 14a), while the other distal moieties of both substrates faced towards R254 (corresponding to R226 in Kce) and most probably form hydrogen bonds between that residue and the β-keto group at a distance of 3.0 Å (acetoacetate) or 2.9 Å (malonate) (Fig. 2g, Supplementary Fig. 14b). While the distal part of KAH in PDB 2Y7F faces towards E14, V87 and F114 (corresponding to I17, P90, F118 in BKACE15), our structures show this space to be occupied by acetyl-CoA. Although there are apparently two conformations how β-keto acids can be positioned in the active site, we assume that upon binding of acetyl-CoA, only one substrate position is plausible to allow the reaction mechanism to work in the forward as well as the backward direction. To start the reaction, a proton must be abstracted from the C2-carbon of MSA. Based on the initial structure 2Y7F, a mechanism was proposed, where R226 as part of catalytic dyad with D231 (corresponding to D259 in BKACE15) deprotonates a water molecule that is bound to the Zn^{2+}. This water in turn was proposed to deprotonate the C2-carbon of KAH [6]. However, we believe that the position of that water will be occupied by the beta-oxo group of MSA similar to the bound acetoacetate and malonate in the crystal structures with and without acetyl-CoA or CoA. Instead, we propose E171 (corresponding to E143 in Kce) to be the residue that deprotonates the MSA substrate. Its carboxyl group has a distance of 3.7 Å to the C2 and can even move closer. It therefore seems the most plausible candidate for stereoselective deprotonation. We performed mutagenesis on that site to validate the role of E171 and observed, that replacing it by leucine abolishes activity with MSA and acetyl-CoA. In contrast, replacing E171 by aspartate, which is still negatively charged but shorter, resulted in reduced but measurable activity (Supplementary Table 4), supporting our hypothesis.

Based on our structural findings we propose a reaction mechanism for the reaction of MSA with acetyl-CoA that starts with the deprotonation of the C2-carbon of MSA by E171 (Supplementary Fig. 15), resulting in an enolate with the negative charge on the oxo-group being stabilized by R254 and Zn^{2+}. The enolate then performs a nucleophilic attack on the carbonyl-group of acetyl-CoA forming a new bond. Subsequently, CoA is eliminated and therefore free to attack at the oxo-group of the original MSA backbone. In a next step, the CoA

ester intermediate dissociates into formyl-CoA and the enolate of acetoacetate, the latter which is re-protonated, likely by E171. The proposed mechanism is in line with the observed positions of acetyl-CoA and acetoacetate, and would also allow the reverse reaction starting from acetoacetate and formyl-CoA, assuming that both acetyl-CoA and formyl-CoA likely bind in the exact same position.

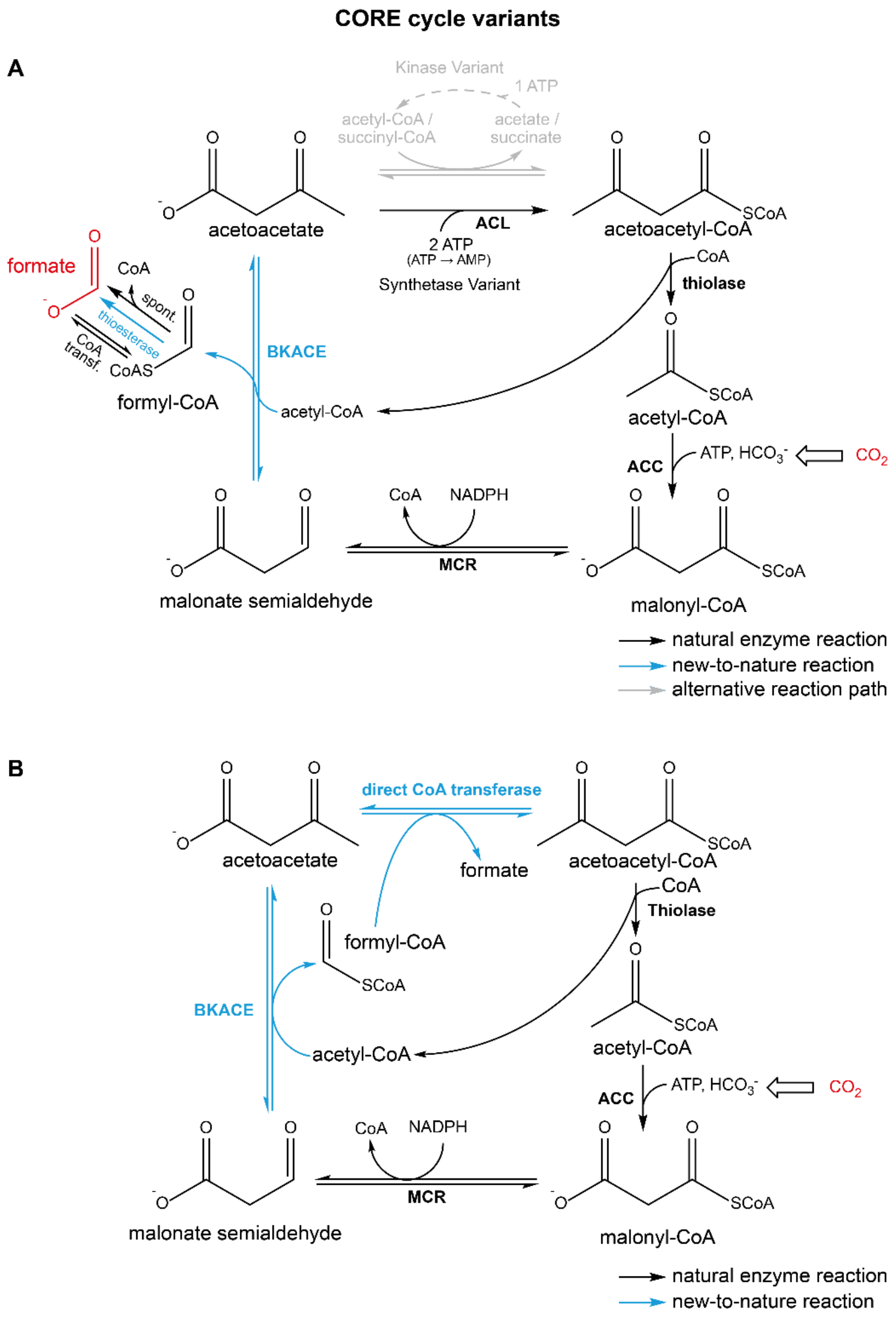

Supplementary Fig. 1. The CORE cycle variants. The CORE cycle is based on a promiscuous reaction by a β-ketoacid cleavage enzyme (BKACE) and can be realized in three variants: via an AMP-forming synthetase/ligase **(A**, black**)**, via a kinase **(A**, grey**)** or via a CoA-transferase **(B)**. Initially, malonyl-CoA is produced from bicarbonate and acetyl-CoA via acetyl-CoA carboxylase (ACC) and subsequently reduced to malonate semialdehyde (MSA) via malonyl-CoA reductase (MCR). The proposed BKACE reaction then condenses MSA with acetyl-CoA to produce formyl-CoA and acetoacetate. In the synthease variant, formyl-CoA is hydrolyzed either spontaneously or via a dedicated/promiscuous thioesterase while acetoacetate is activated to acetoacetyl-CoA via acetoacetyl-CoA ligase (ACL) with concomitant release of AMP (resulting in a cost of 2 ATP for this reaction to regenerate ATP from AMP). In the kinase variant, acetoacetate is activated by another CoA-thioester serving as CoA-donor, which is regenerated by a kinase at the cost of 1 ATP. In the CoA-transferase variant, formyl-CoA serves as a CoA donor to conserve energy and activate acetoacetate directly to acetoacetyl-CoA, releasing formate. Finally, two molecules of acetyl-CoA are regenerated from acetoacetyl-CoA via β-ketothiolase. The max-min driving force (MDF) [7–9] values for each variant are as follows: 8.75 kJ/mol for the direct CoA-transferase variant; 5.69 kJ/mol for the Kinase variant (via succinyl-CoA synthetase); and 16.64 kJ/mol for the Synthetase/Ligase variant.

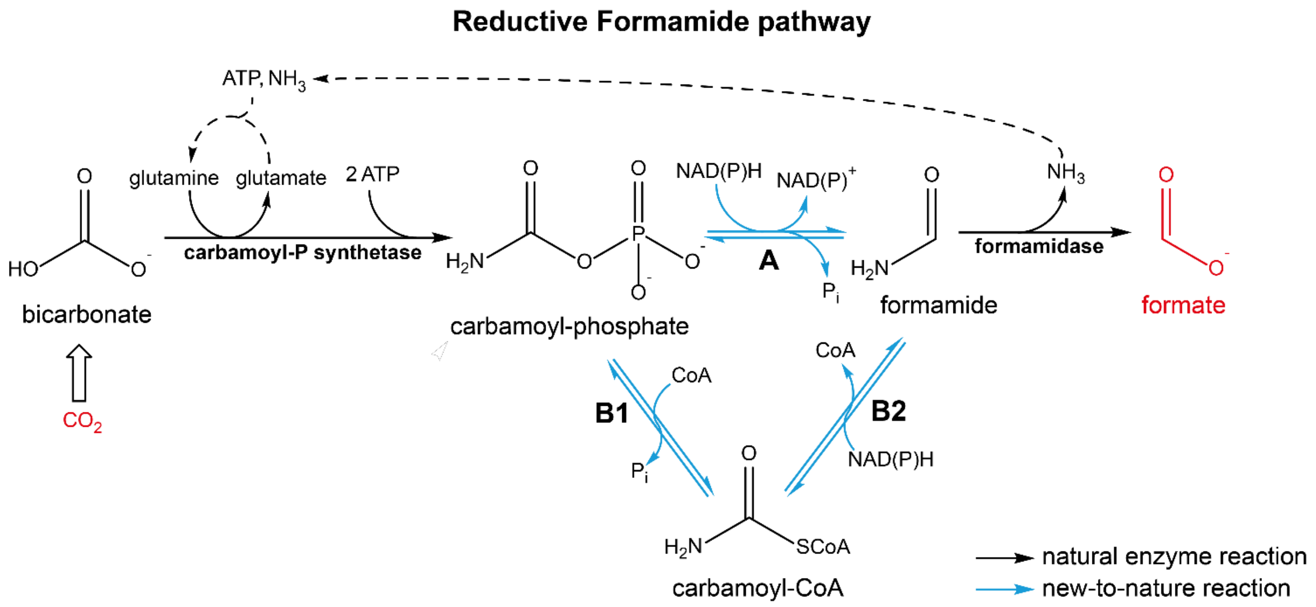

Supplementary Fig. 2. The reductive formamide pathway. Initially, carbamoyl-phosphate (CP) is produced from bicarbonate and ammonia (derived from glutamine) in an irreversible reaction via carbamoyl-phosphate synthetase at the overall cost of 3 ATP (including ATP for the conversion of glutamate to glutamine as NH_3 donor). For the subsequent new-to-nature reduction reaction, two routes are possible: Either in a single step via a phosphorylating aldehyde dehydrogenase (reaction A, e.g. EC [enzyme commission number] 1.2.1.11, 1.2.1.12, 1.2.1.38 or 1.2.1.41) or in two steps with a carbamoyl-CoA intermediate, involving a phosphate acyltransferase (reaction B1; e.g. EC 2.3.1.8, 2.3.1.19 or 2.3.1.222) together with an acylating aldehyde dehydrogenase (B2; e.g. PduP-like enzymes). Finally, formamide is converted to formate by formamidase, regenerating NH_3.

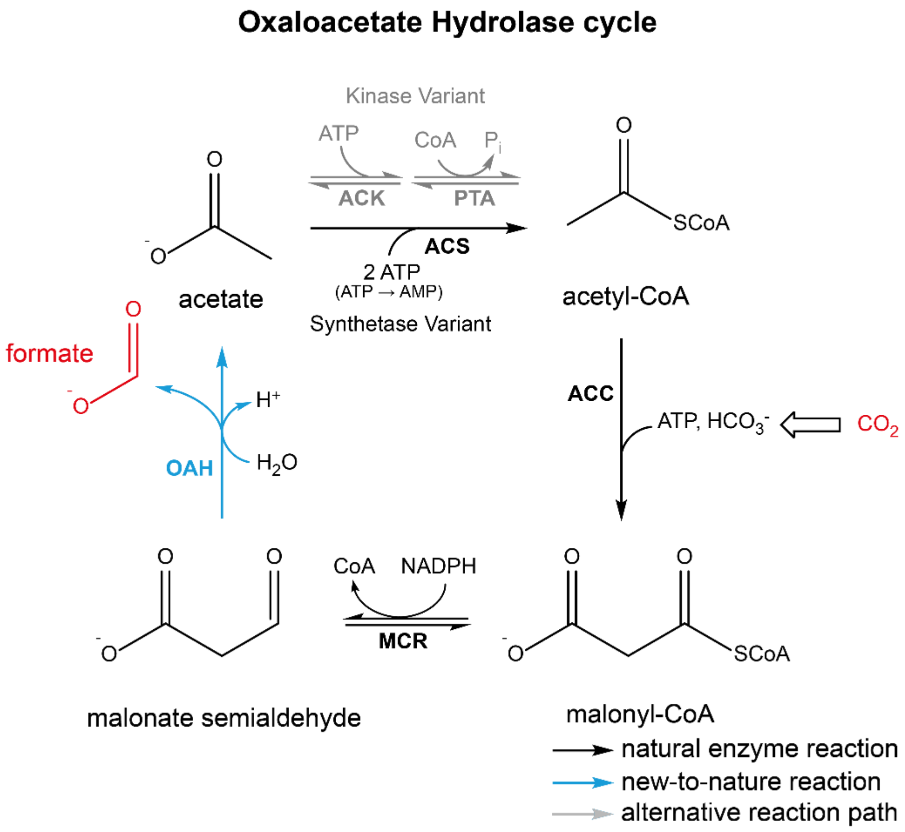

Supplementary Fig. 3. The oxaloacetate hydrolase (OAH) cycle. Initially, malonyl-CoA is produced from bicarbonate and acetyl-CoA via acetyl-CoA carboxylase (ACC) and subsequently reduced to malonate semialdehyde (MSA) by malonyl-CoA reductase (MCR). The key novel reaction then irreversibly hydrolyzes MSA to formate and acetate based on a proposed promiscuity of oxaloacetate hydrolase (OAH, EC 3.7.1.1). Finally, the regeneration of acetyl-CoA from acetate occurs either directly via acetyl-CoA synthetase (ACS) at the cost of 2 ATP, or in two steps via acetate kinase (ACK) and phosphotransacetylase (PA) at the cost of 1 ATP.

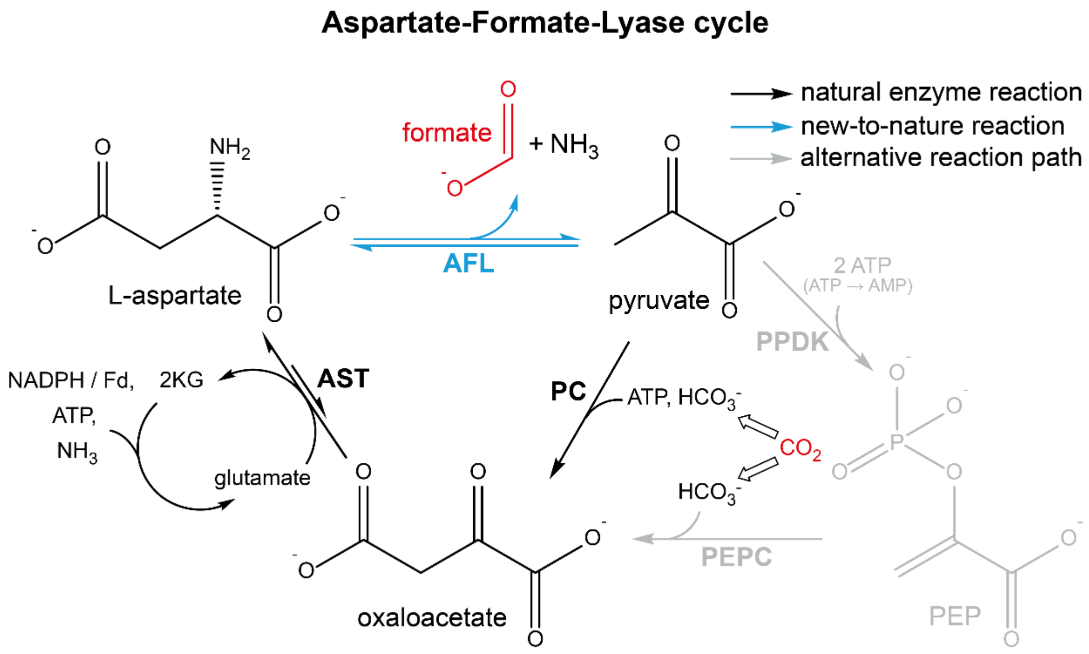

Supplementary Fig. 4. The aspartate-formate-lyase (AFL) cycle. Initially, oxaloacetate is produced from pyruvate by incorporation of bicarbonate either via pyruvate carboxylase (PC) at the cost of 1 ATP or via pyruvate-phosphate-dikinase (PPDK, also commonly referred to as "PEP synthetase") and PEP carboxylase (PEPC) at the cost of 2 ATP. Oxaloacetate is subsequently converted to L-aspartate via aspartate transaminase (AST), requiring glutamate as NH_3 donor that is regenerated either by NAD(P)H-dependent glutamate dehydrogenase (in bacteria; no ATP required) or by glutamate synthase together with glutamine synthetase (in plants; ferredoxin as reductant; requires 1 ATP). The key novel reaction finally cleaves L-aspartate into formate, NH_3 and pyruvate based on the reported activity of an engineered aspartate-formate lyase (AFL). AFL was previously engineered by changing the substrate specificity of tyrosine-phenol-lyase (TPL, EC 4.1.99.2) to accept dicarboxylic amino acids for the catalyzed β-elimination reaction [4]. 2KG: α-ketoglutarate, PEP: phosphoenolpyruvate.

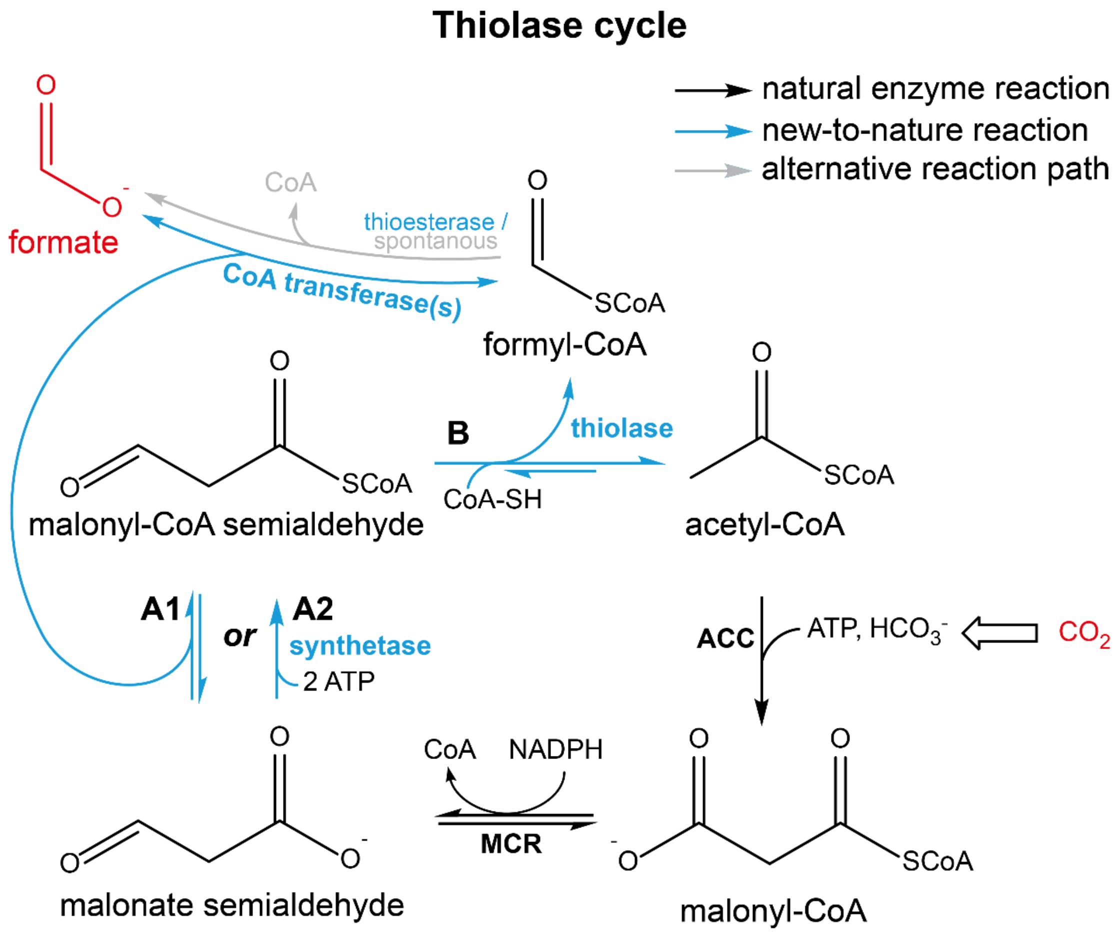

Supplementary Fig. 5. The thiolase cycle. Initially, malonyl-CoA is produced from bicarbonate and acetyl-CoA via acetyl-CoA carboxylase (ACC) and subsequently reduced to malonate semialdehyde (MSA) by malonyl-CoA reductase (MCR). MSA is then activated to malonyl-CoA semialdehyde in a new-to-nature reaction either via an energy-conserving CoA transferase (reaction A1, e.g. ECs 2.8.3.5, 2.8.3.8 or 2.8.3.9) or via a synthetase at the cost of 2 ATP (reaction A2, EC 6.2.1.16). The key novel reaction is a thiolytic cleavage reaction of malonyl-CoA semialdehyde into formyl-CoA and acetyl-CoA (reaction B, e.g. ECs 2.3.1.9 or 2.3.1.16). Finally, formyl-CoA is converted to formate, either via the CoA transferase (A1), via a new-to-nature thioesterase reaction or by spontaneous hydrolysis.

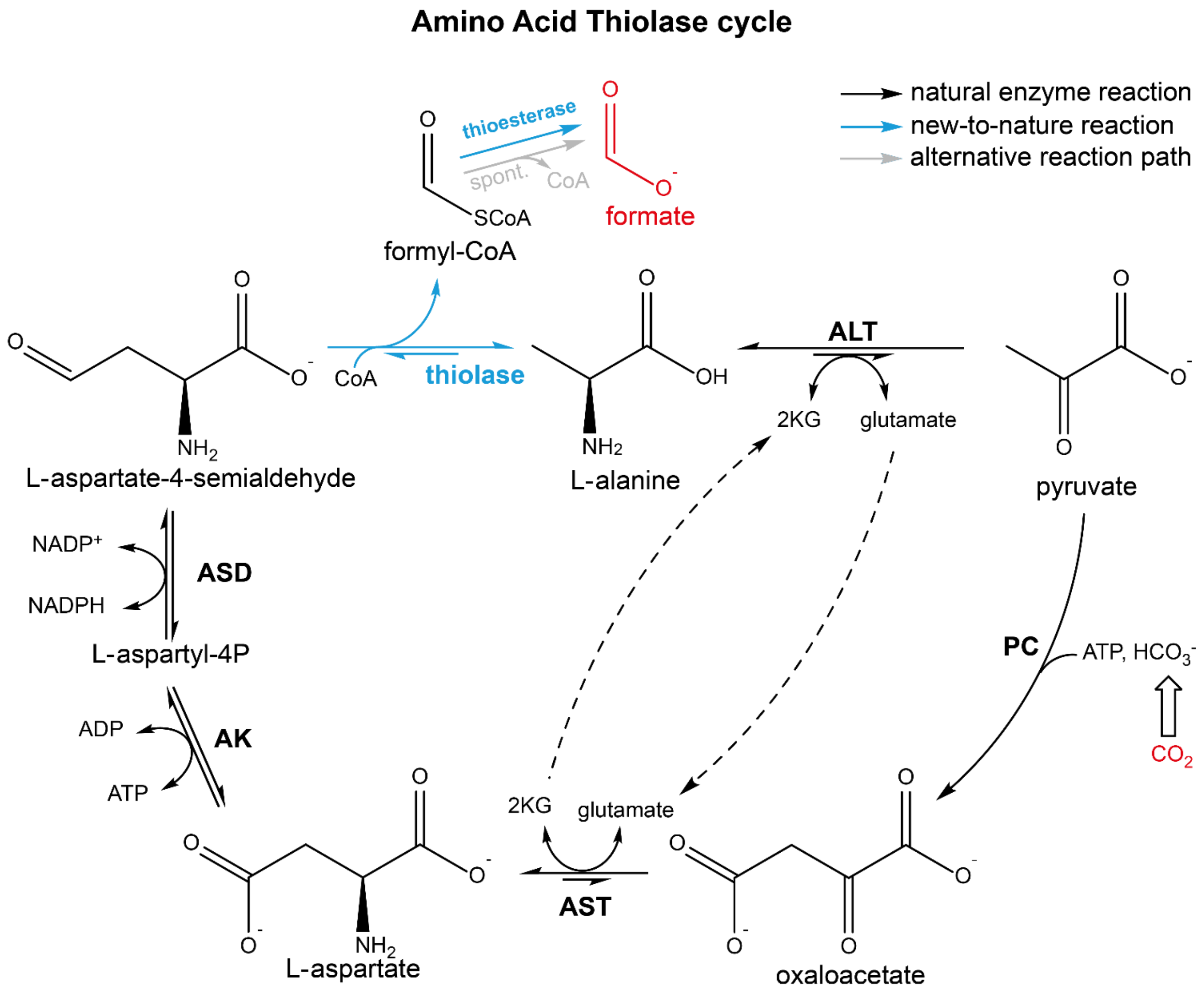

Supplementary Fig. 6. The amino acid thiolase cycle. Initially, oxaloacetate is produced from bicarbonate and pyruvate via pyruvate carboxylase (PC) at the cost of 1 ATP. Oxaloacetate is subsequently converted to L-aspartate via aspartate transaminase (AST) and glutamate as NH_3 donor. Activation of L-aspartate via aspartate kinase (AK) and subsequent oxidation via aspartate-semialdehyde dehydrogenase (ASD) produces L-aspartate-4-semialdehyde. In the key novel reaction, it is then thiolytically cleaved into L-alanine and formyl-CoA via a PLP-dependent thiolase (EC 2.3.1.263). While L-alanine is converted to pyruvate via alanine transaminase (ALT) regenerating glutamate, formyl-CoA is converted to formate either via a new-to-nature thioesterase reaction or by spontaneous hydrolysis.

Formyl-Pyruvate Hydrolase cycle

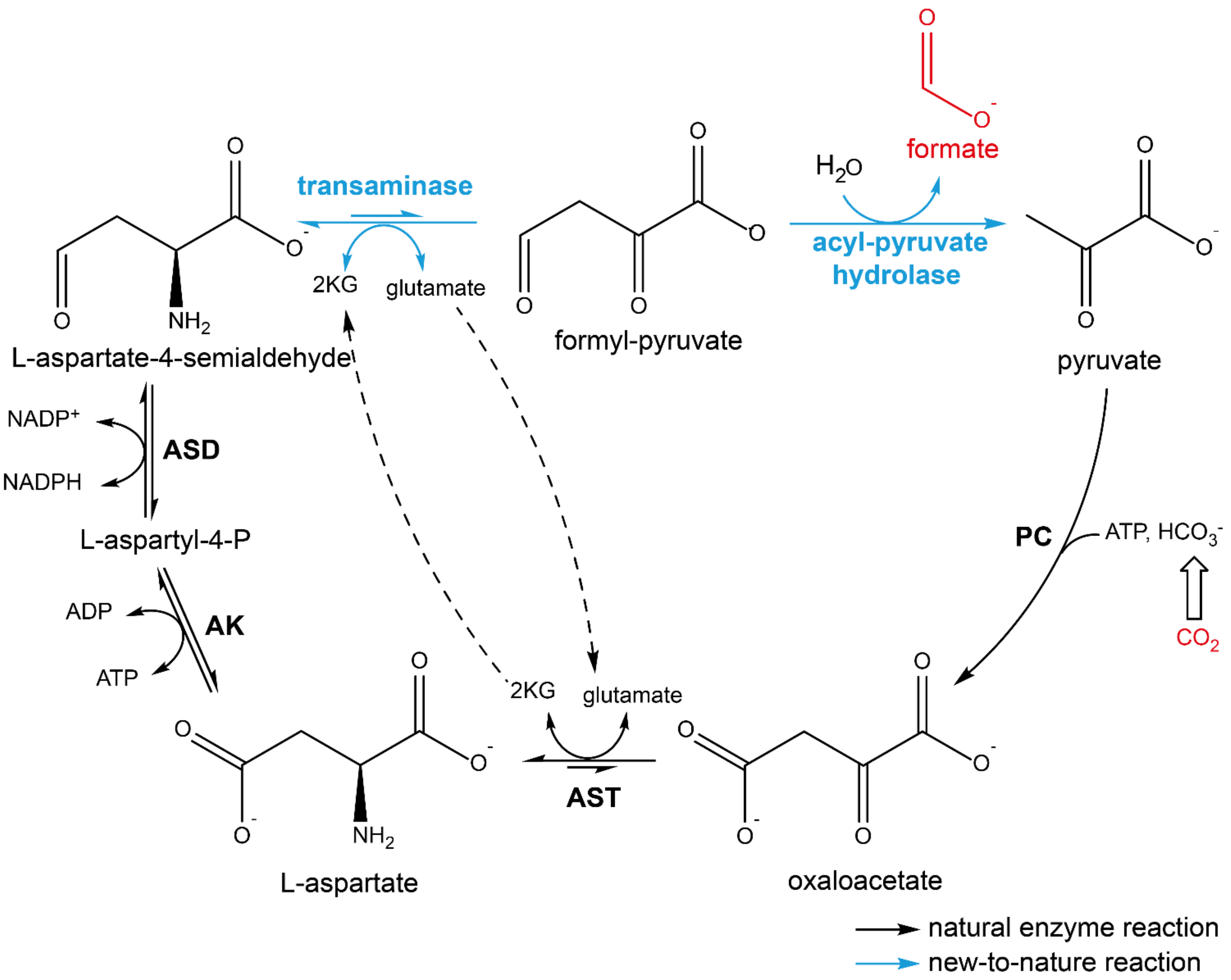

Supplementary Fig. 7. The formyl-pyruvate hydrolase cycle. Initially, oxaloacetate is produced from bicarbonate and pyruvate via pyruvate carboxylase (PC) at the cost of 1 ATP. Oxaloacetate is subsequently converted to L-aspartate via aspartate transaminase (AST) and glutamate as NH_3 donor. Activation of L-aspartate via aspartate kinase (AK) and subsequent oxidation via aspartate-semialdehyde dehydrogenase (ASD) produces L-aspartate-4-semialdehyde. In a new-to-nature reaction, a transaminase converts it to formyl-pyruvate thereby regenerating glutamate. The key novel reaction finally cleaves it to formate and pyruvate via an acyl-pyruvate hydrolase (EC 3.7.1.5).

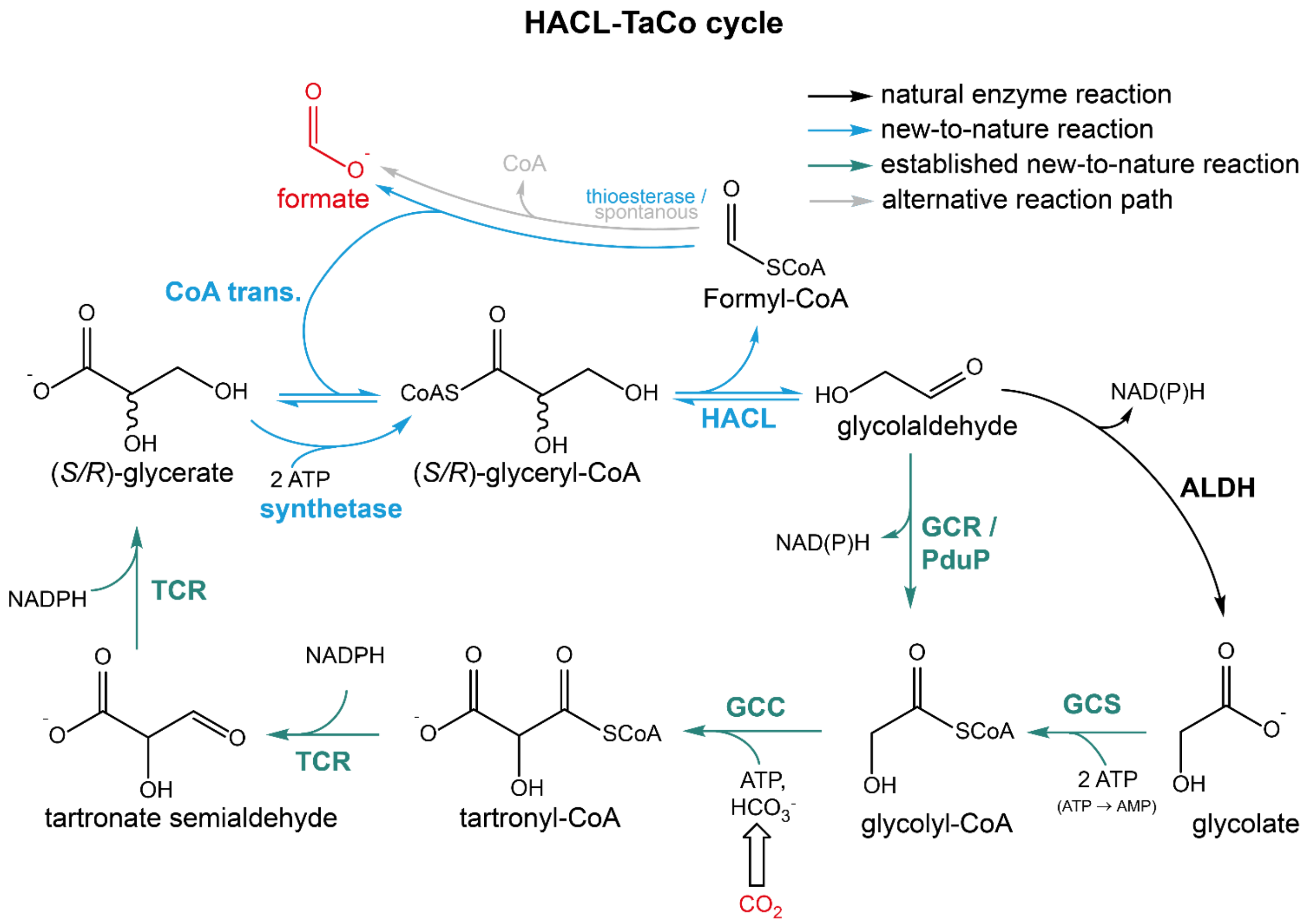

Supplementary Fig. 8. The HACL-TaCo cycle. Initially, tartronyl-CoA is produced from bicarbonate and glycolyl-CoA via glycolyl-CoA carboxylase (GCC) and subsequently reduced two times to tartronate semialdehyde and further to glycerate via tartronyl-CoA reductase (TCR). In a new-to-nature reaction, glycerate is then activated to glyceryl-CoA either via a synthetase at the cost of 2 ATP or via a CoA transferase. In a second new-to-nature reaction, glyceryl-CoA is cleaved into glycolaldehyde and formyl-CoA via 2-hydroxyacyl-CoA lyase (HACL). Finally, formate is produced from formyl-CoA either via the afore mentioned CoA transferase, via a new-to-nature thioesterase reaction or by spontaneous hydrolysis. Closing of the cycle and regeneration of glycolyl-CoA occurs either by the direct reduction of glycolaldehyde or in two steps with a glycolate intermediate. The direct reduction occurs via glycolyl-CoA reductase (GCR) or via CoA-acylating propionaldehyde reductase (PduP). The two-step route occurs via glycolaldehyde dehydrogenase (ALDH) and glycolyl-CoA synthetase (GCS) at the cost of 2 ATP. GCS, GCC and TCR have already been engineered to catalyze the desired reactions as part of the tartronyl-CoA (TaCo) pathway [10,11]. GCR has also already been engineered from *Rhodopseudomonas palustris* PduP [10].

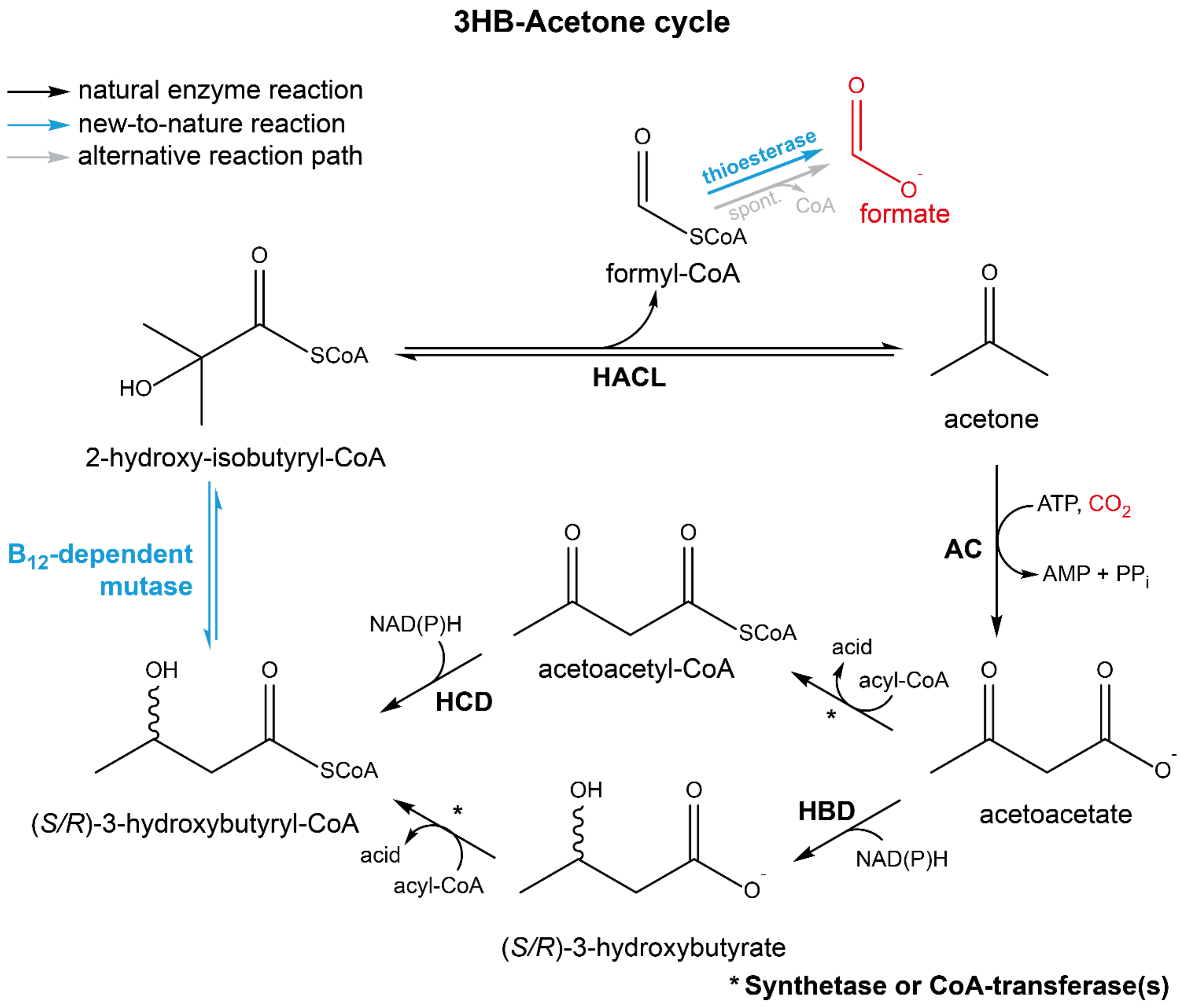

Supplementary Fig. 9. The 3-hydroxybutyrate-acetone cycle. Initially, acetoacetate is produced from bicarbonate and acetone via acetone carboxylase (AC) at the cost of 2 ATP. For the subsequent conversion of acetoacetate to 3-hydroxybutyryl-CoA, two routes are possible: In the first route, a reduction via 3-hydroxybutyrate dehydrogenase (HBD) is followed by an activation either via a synthetase at the cost of 1 ATP or via a CoA-transferase. In the second route, the activation comes first, followed by a reduction via 3-hydroxybutyryl-CoA dehydrogenase (HCD). In a new-to-nature reaction, 3-hydroxybutyryl-CoA is first converted to 2-hydroxy-isobutyryl-CoA via a B_{12}-dependent mutase, and then cleaved into acetone and formyl-CoA via 2-hydroxyacyl-CoA lyase (HACL) [12]. Candidates for the mutase reaction have been demonstrated before for the (*S*-)-isomer [13–16] and the (*R*)-isomer [17,18]. Finally, formate is produced from formyl-CoA either via a new to-nature thioesterase reaction or by spontaneous hydrolysis.

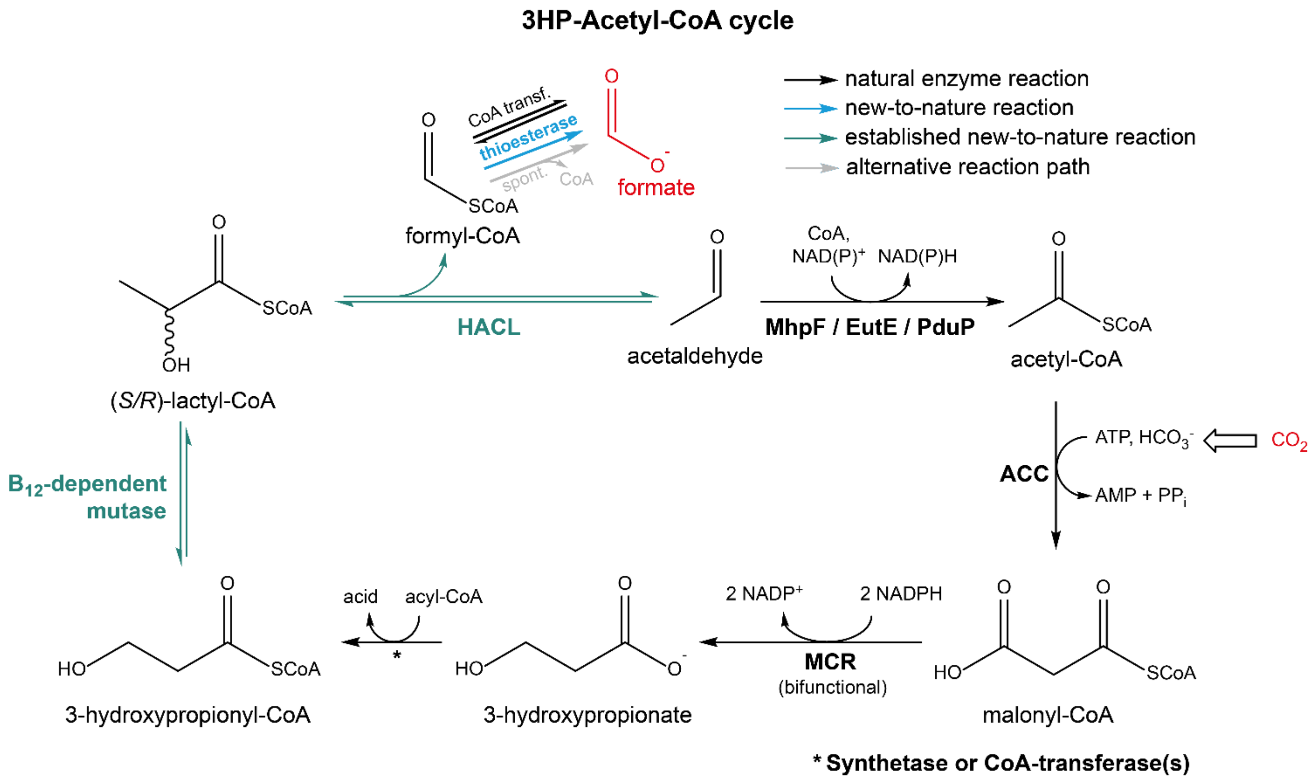

Supplementary Fig. 10. The 3-hydroxypropionate-acetyl-CoA cycle. Initially, malonyl-CoA is produced from bicarbonate and acetyl-CoA via acetyl-CoA carboxylase (ACC) at the cost of 1 ATP and subsequently reduced two times to 3-hydroxypropionate via a bifunctional malonyl-CoA reductase (MCR). Subsequently, 3-hydroxyproionate is activated to its corresponding CoA ester either via an energy-conserving CoA transferase or via a synthetase at the cost of 2 ATP. In two novel reactions, a B_{12}-dependent mutase produces (*S*)- or (*R*)-lactyl-CoA and a 2-hydroxyacyl-CoA lyase (HACL) cleaves it into formyl-CoA and acetaldehyde [19]. For the mutase reaction, an enzyme candidate has recently been identified and characterized (Schulz-Mirbach *et al.* – manuscript in revision). Regeneration of acetyl-CoA from acetaldehyde occurs via acetylating acetaldehyde dehydrogenae (MhpF / EutE) or via CoA-acylating propionaldehyde reductase (PduP). Finally, formyl-CoA is converted to formate, either via the afore mentioned CoA transferase, via a new-to-nature thioesterase reaction or by spontaneous hydrolysis.

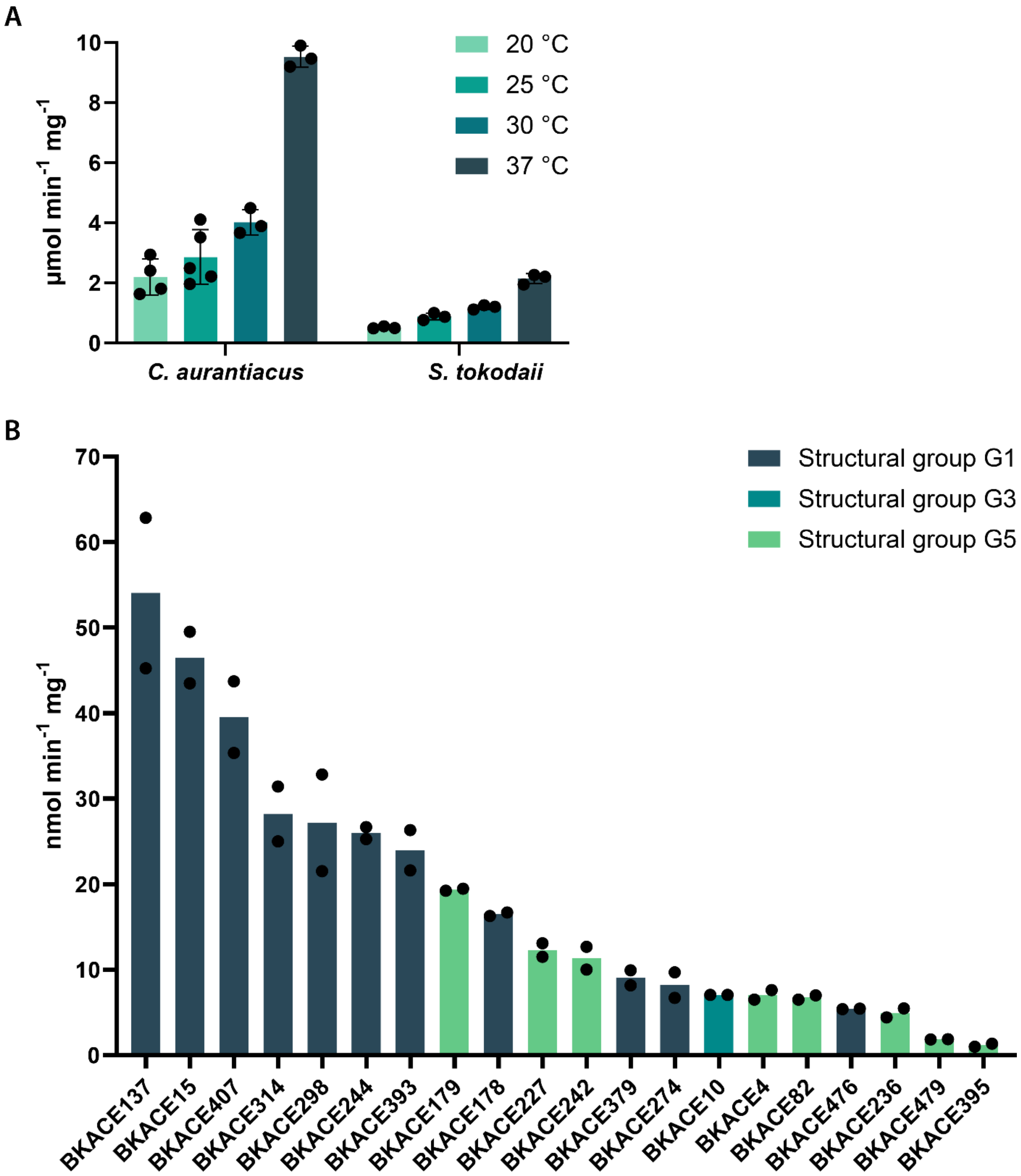

Supplementary Fig. 11. Specific activities for MCR and BKACE candidates. A) Temperature dependence of Malonyl-CoA Reductase. Specific activities were measured at different temperatures. *C. aurantiacus*: Malonyl-CoA Reductase C-domain from *Chloroflexus aurantiacus* (MCR); *S. tokodaii*: Malonyl-CoA Reductase from *S. tokodaii* (Mcr_St). Measurements were done in triplicates. Mean and individual datapoints are shown. Error bars indicate standard deviations. **B)** Specific activities for the 20 best-performing BKACE candidates from the high-throughput screen. The color code indicates different structural groups of the BKACE family that were described before [5]. The best-performing candidates identified here all belong to structural group G1 that was reported to accept β-keto acids with apolar and non-charged terminal moieties. MSA that only has a hydrogen atom as moiety fits well into that group which may explain the high abundance in our results. Activities were determined in presence of 250 µM acetyl-CoA and 1.2 mM MSA in 50 mM Tris-HCl (pH 7.5) by LC-MS/MS analysis. Measurements were done in duplicates, mean and individual data points are shown. Source data are provided as a Source Data file.

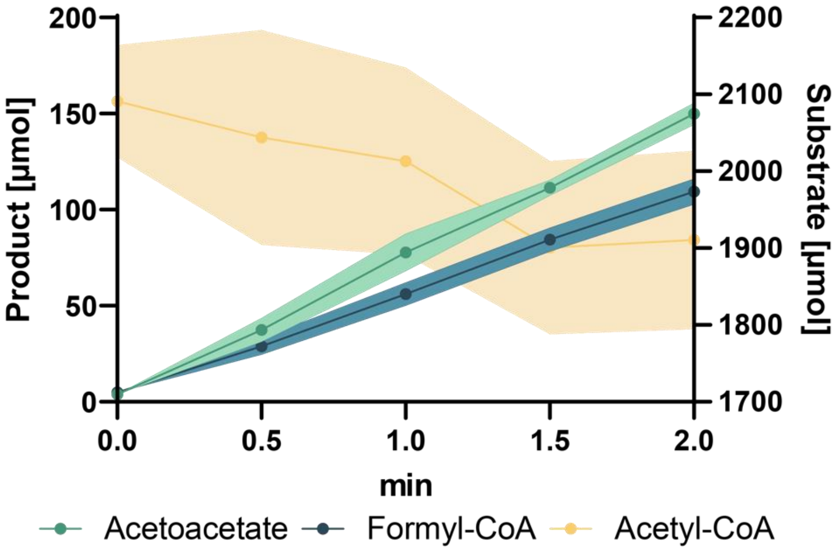

Supplementary Fig. 12. Reaction kinetics for the optimized BKACE screen. Substrate consumption and product formation for BKACE15 were measured with 4 mM MSA and 1 mM acetyl-CoA. Substrate consumption of acetyl-CoA is plotted on the right axis, product formation of acetoacetate and formyl-CoA on the left axis. Shaded areas reflect standard deviations. Lower titers of formyl-CoA compared to acetoacetate are due to spontaneous hydrolysis. Source data are provided as a Source Data file.

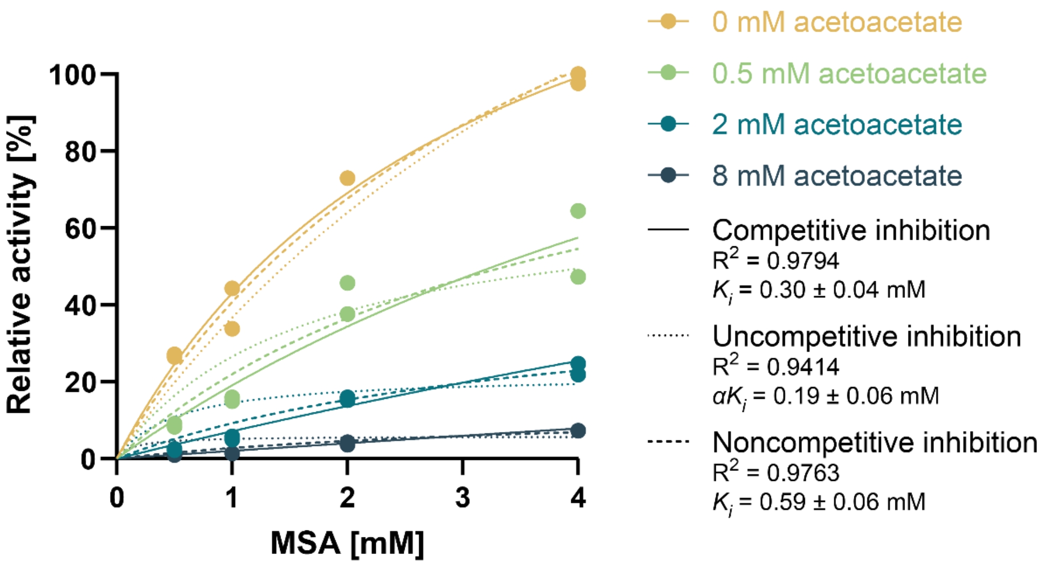

Supplementary Fig. 13. Inhibition type modelling for the product inhibition of BKACE15 by acetoacetate. Different Michaelis-Menten inhibition types were modelled, with competitive and noncompetitive inhibition found to be the types that describe the data best. The reversible character of the catalyzed reaction further supports the existence of a competitive inhibition mechanism due to definitive binding of acetoacetate as substrate for the reverse reaction. We note that an additional effect from a noncompetitive inhibition at an allosteric site is still possible. Measurements were performed in duplicates. K_i and αK_i represent the inhibition constants for the different types. Data were visualized and fitted using GraphPad Prism v10. Source data are provided as a Source Data file.

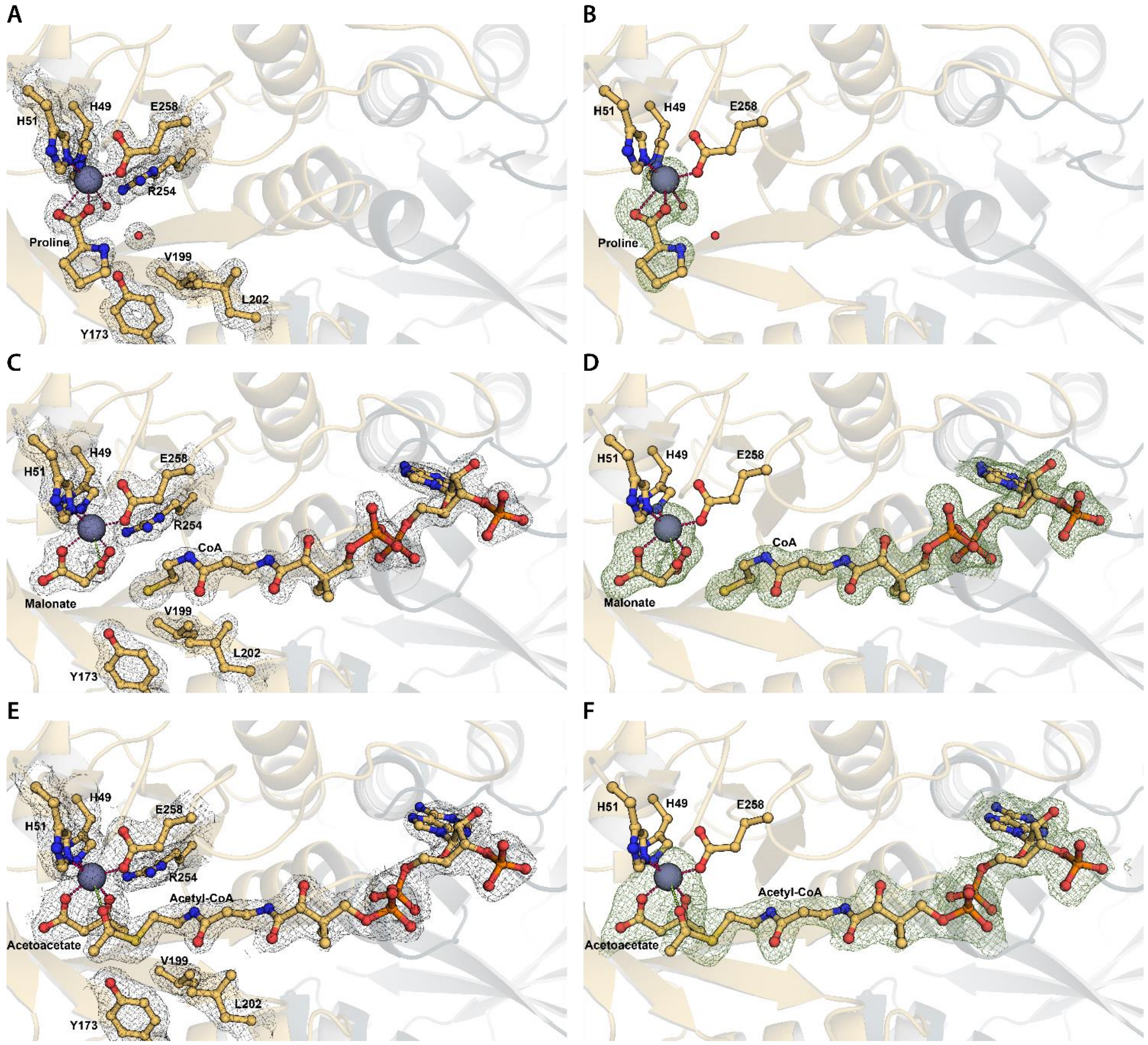

Supplementary Fig. 14. Electron density maps for KCE15. Left panels: 2Fo-Fc maps for residues surrounding the Zn^{2+} binding site. Right panels: Fo-Fc simulated annealing omit maps (Fo-Fc) for bound ligands. **A)** Electron density for active site residues (PDB 8RIO) surrounding Zn^{2+} and proline at 1.20 σ. **B)** Omit map (PDB 8RIO) for Zn^{2+} and proline at 2.50 σ. **C)** Electron density for active site residues (PDB 8RIP) surrounding Zn^{2+}, malonate, and CoA at 1.50 σ. **D)** Omit map (PDB 8RIP) for Zn^{2+}, malonate, and CoA at 2.25 σ. **E)** Electron density for active site residues (PDB 9HNF) surrounding Zn^{2+}, acetoacetate, and acetyl-CoA at 1.2 σ. **F)** Omit map (PDB 9HNF) for Zn^{2+}, acetoacetate, and acetyl-CoA at 2.0 σ. Coordinating interactions for Zn^{2+} are highlighted in pink. Two more distant contacts (2.8 Å) between Zn^{2+} and a water molecule in panels C & D as well as between Zn^{2+} and the thioester oxygen of acetyl-CoA in panels E & F are highlighted in lime green.

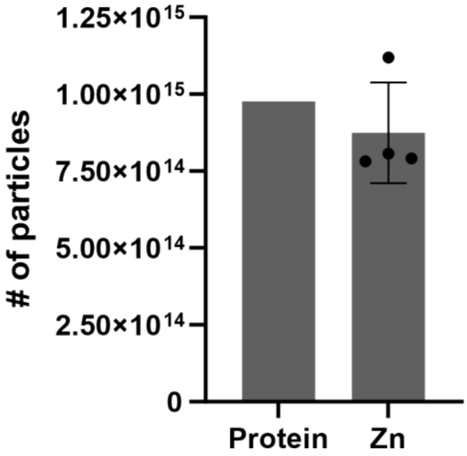

Supplementary Fig. 15. Inductively coupled plasma optical emission spectroscopy (ICP-OES) of BKACE15. Abundance of Zn atoms is represented as mean value of 4 technical replicates. The abundance of protein particles was calculated based on its molecular weight. The data demonstrate a ratio of 0.9 ± 0.1 Zn atoms per subunit of BKACE15, confirming Zn as the corresponding metal bound in the active site. Source data are provided as a Source Data file.

A

```
BKACE15    MSLNGKVIIT CAVTGAIHTP SMSPYLPVSA SEITDAAIGA AEAGAAVIHL    50
Kce        ---MEPLILT AAITGAETTR ADQPNLPITP EEQAKEAKAC FEAGARVIHL    47
                 :*:* .*:***  *  : .* **::. .* :. * ..  **** ****

BKACE15    HARHEGDGSP DQSVEAFNPI LGVIKQ-ASD AVLNITTGGA PTMSIAERIQ    99
Kce        HIR-EDDGRP SQRLDRFQEA ISAIREVVPE IIIQISTGGA VGESFDKRLA    96
           * * *.** * .* :: *:   :..*.: ..:  :::*:****    *: :*:

BKACE15    PAQHYRPELA SLNMGTMNFG LFPMLNRYES QLKHQWERNY LGNKDIIFRN   149
Kce        PLA-LKPEMA TLNAGTLNFG ---------- ---------- ----DDIFIN   121
           *    .**:* :** **:***                           * ** *

BKACE15    TFGDVEHVMT TLGAGGTRFE FECYDTSHLY NLKHFYDRGL V-KGPLFIQT   198
Kce        HPADIIRLAE AFKQYNVVPE VEVYESGMVD AVARLIKKGI ITQNPLHIQF   171
             .*: .:   ::   ..  * .* *::. :   : .: ..*: : :.**.**

BKACE15    VFGLMGGIGA HPDDVLHMKR TADRLFGQDY RWSVLGAGRN QLNIAAMSAA   248
Kce        VLGVPGGMSG KPKNLMYMME HLKEEIPTA- TWAVAGIGRW HIPTSLIAMV   220
           *:*: **:.. :*.::::*     .  :      *:* * **. ::  : :: .

BKACE15    MGGHVRVGLE DNLWAGKGRL AETNAQQVRA ARQIVEGLGL EVATPAEARE   298
Kce        TGGHIRCGFE DNIFYHKGVI AESNAQLVAR LARIAKEIGR PLATPEQARE   270
            ***:* *:* **::  ** : **:*** *     .*.: :*   :*** :***

BKACE15    LLALKGGDQV NF                                            310
Kce        ILALNK---- --                                            276
           :***:
```

B

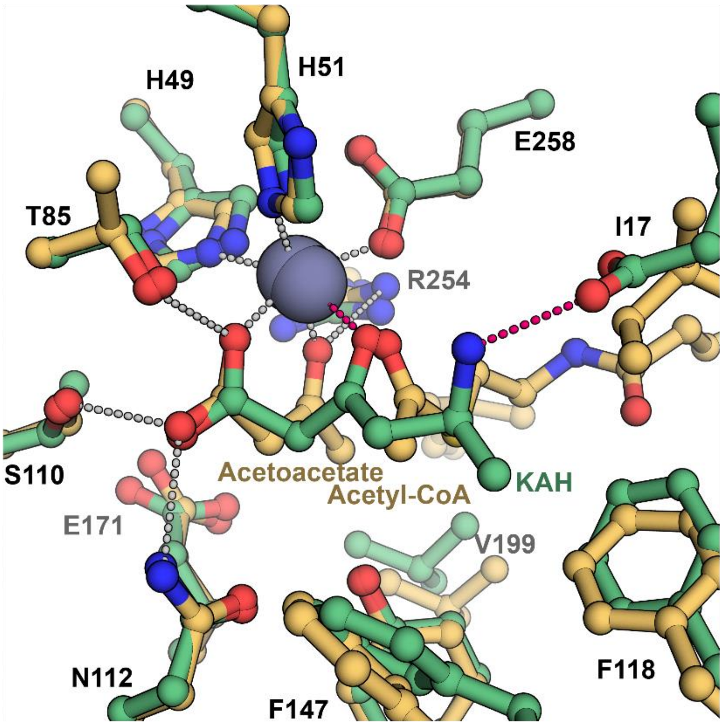

Supplementary Fig. 16. Comparison of BKACE15 and Kce. A) Protein sequence alignment of BKACE15 (Uniprot A1B802) and Kce from *Candidatus* Cloacimonas acidaminovorans [6] (Uniprot B0VHH0) for comparison with the structures (PDB 9HNF and 2Y7F). Asterisks (*) indicate completely conserved residues. Colons (:) indicate conservation between groups of highly similar properties (scoring > 0.5 in the Gonnet PAM 250 matrix). Periods (.) indicate conservation between groups of weak similaraty (scoring ≤ 0.5 in the Gonnet PAM 250 matrix). The alignment was produced with MUSCLE 3.8 [20]. **B)** Overlay of the active sites of BKACE15 and Kce. BKACE15 (PDB 9HNF) is shown in yellow; Kce (PDB 2Y7F) is shown in green. Labeling of the substrates is shown in the color of the corresponding structure; labeling of active site residues corresponds to BKACE15. Labeling of foreground residues is shown in black, labeling of background residues in dark grey. Polar interactions are indicated by grey dashes; polar interactions that are specific to KAH binding are indicated in pink. Zn^{2+} is shown in metal grey. KAH: 3-keto-5-aminohexanoate.

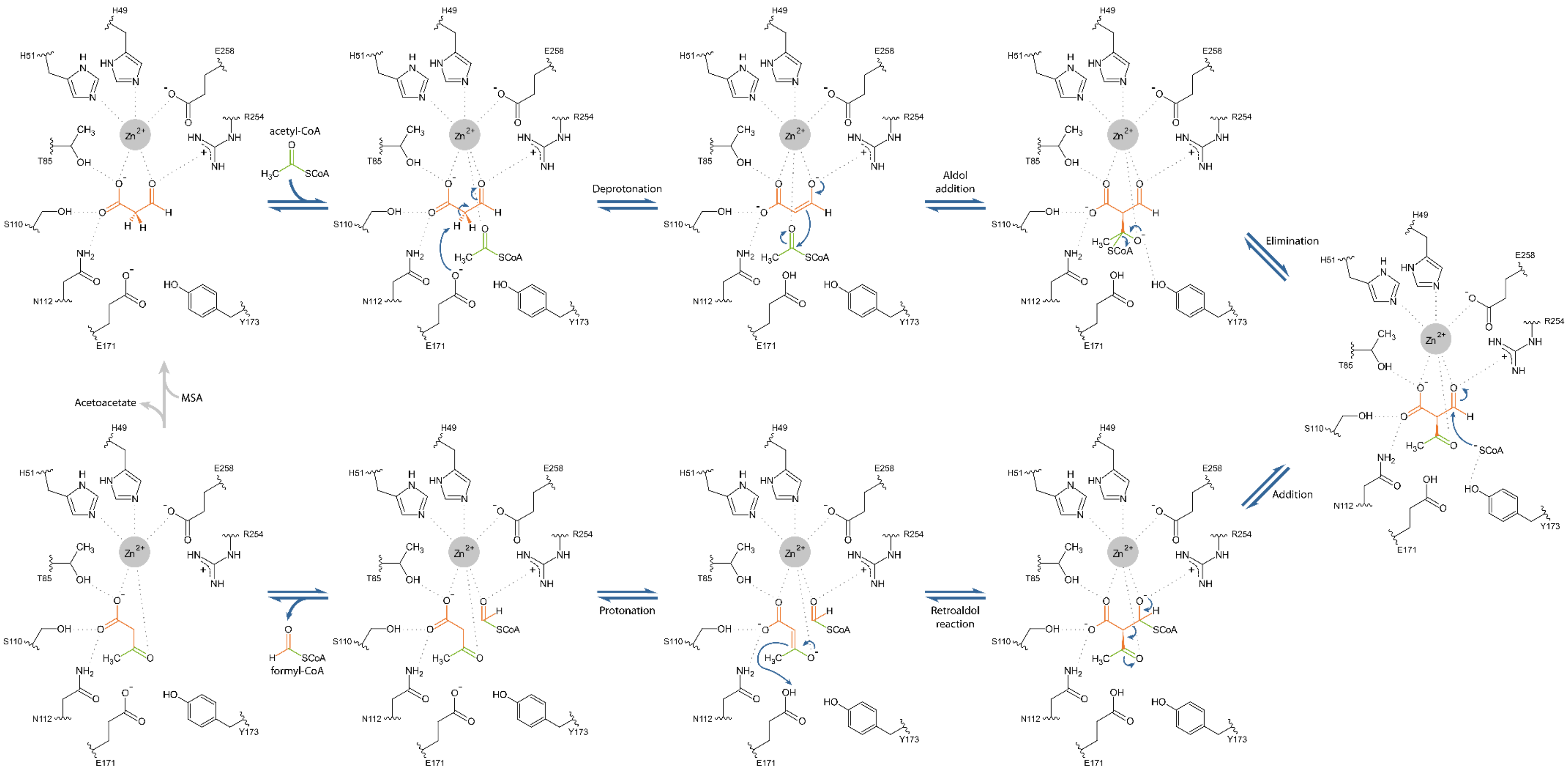

Supplementary Fig. 17. Proposed mechanism for the BKACE reaction. The scheme depicts the reaction sequence starting from MSA and acetyl-CoA. MSA is bound first. Acetyl-CoA binds second and the reaction mechanism is initiated by deprotonation at the C2-carbon of MSA by E171. The formed enolate then performs a nucleophilic attack on the carbonyl of acetyl-CoA forming a high-energy intermediate. After the elimination of CoA and it re-attacking at the aldehyde carbon, the CoA ester dissociates and releases formyl-CoA. The remaining enolate is re-protonated to form acetoacetate. Next, formyl-CoA is released before acetoacetate can leave to make space for another round of catalysis (grey arrow). Acetoacetate as the substrate for the back reaction (with formyl-CoA) can act as an inhibitor and lower the catalytic rate with acetyl-CoA-CoA and MSA. Orange highlights the molecule backbone of MSA, green highlights the molecule backbone of acetyl-CoA. Dotted lines reflect hydrogen bonds and salt bridges.

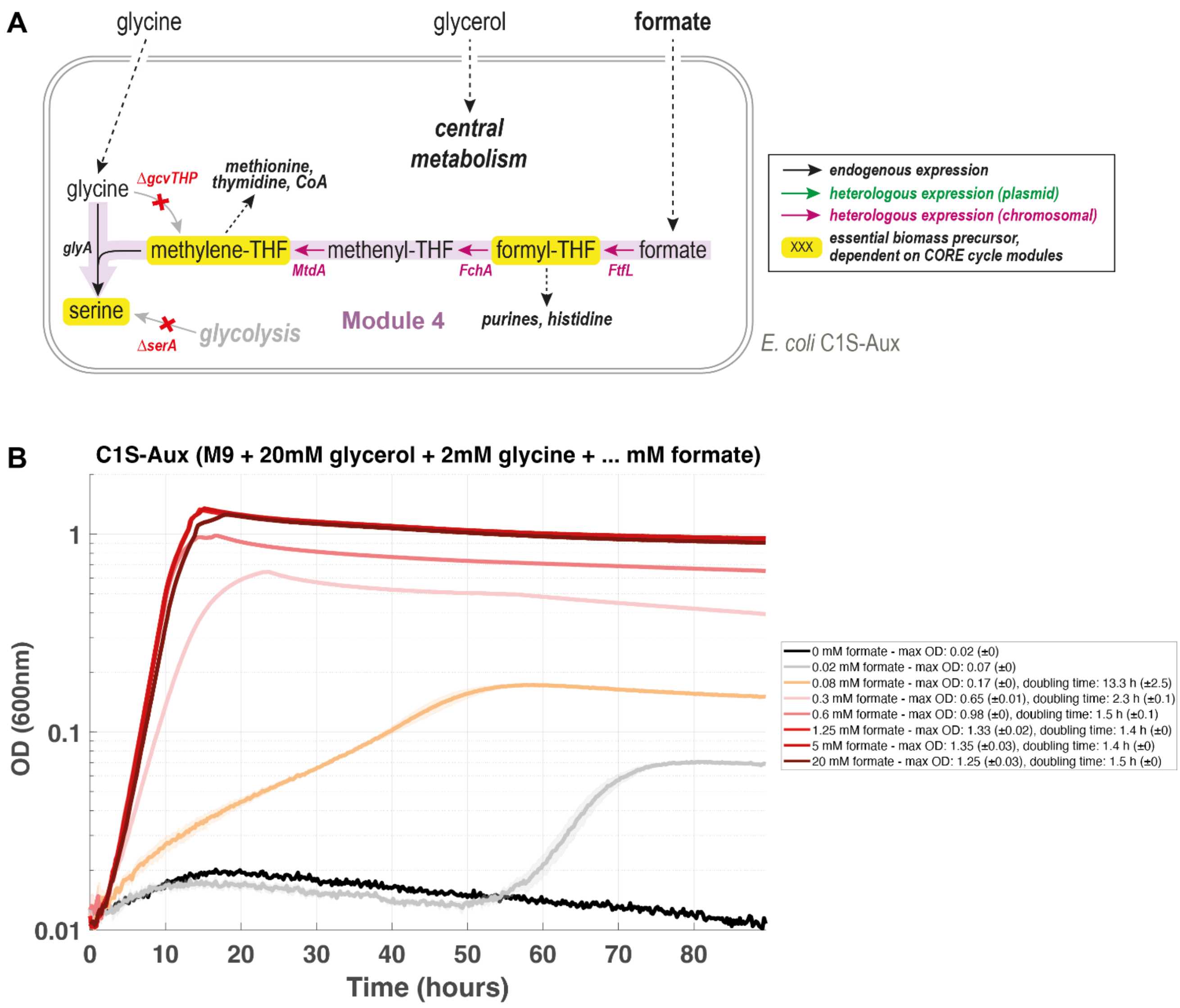

Supplementary Fig. 18. Growth response of the C1S-Aux strain to varying concentrations of formate (sodium formate) supplied in the medium. A) Schematic summarizing the selection. **B)** Growth was assayed in minimal medium with glycerol as main carbon source (M9 + 20 mM glycerol + 2 mM glycine + varying formate concentrations) in a plate reader in 96-well format. Y-axis values represent OD600 normalized to a path length of 1 cm ("cuvette OD"). Curves represent the mean of technical triplicates, with shaded areas representing standard deviation. Doubling times estimated in the exponential growth phase are indicated in the legend (no doubling time was estimated for growth curves remaining below OD 0.1). Source data are provided as a Source Data file.

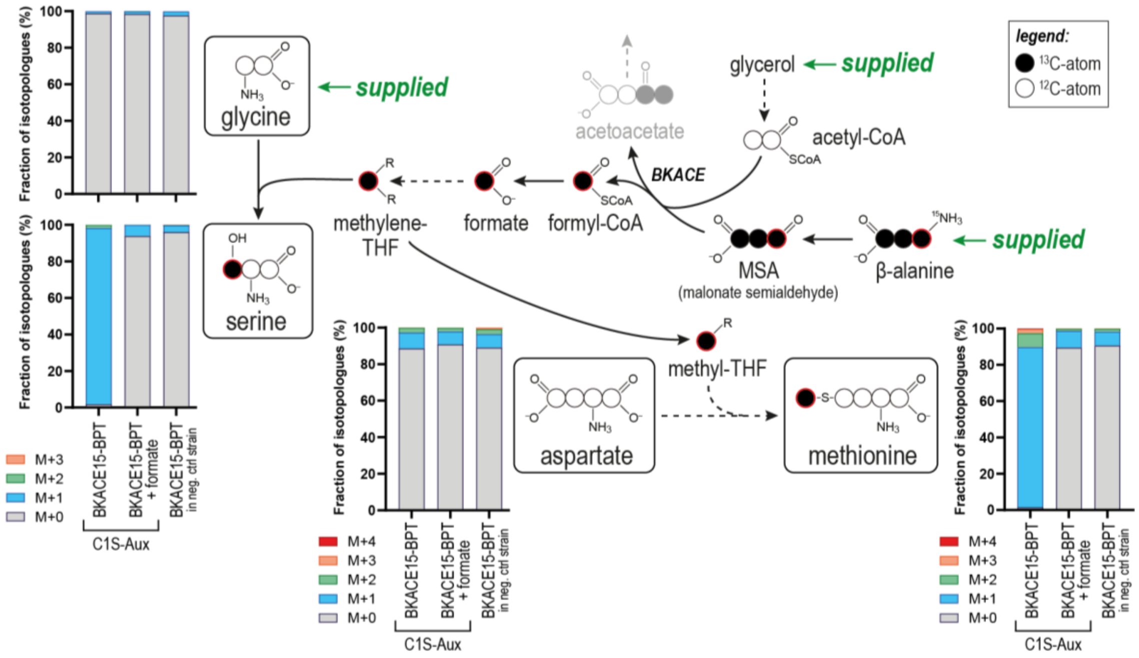

Supplementary Fig. 19. ^{13}C-tracing unequivocally confirms operation of the CORE cycle modules 2 and 4 in growing *E. coli*. Shown is the predicted labeling pattern and observed isotopologue distribution for selected protein-derived amino acids. The selection strain (C1S-Aux + pZ-BKACE15-BPT) was cultivated in minimal medium containing fully labeled $^{13}C_3$-^{15}N-β-alanine and otherwise naturally labeled carbon sources (M9 + 20 mM glycerol + 2 mM glycine + 10 mM $^{13}C_3$-^{15}N-β-alanine). If formate is generated via the CORE cycle module 4 (BKACE15), serine is expected to be single-labeled since its terminal hydroxyl-carbon is derived from formate, ultimately originating from ^{13}C-β-alanine. Similarly, methionine is expected to show single ^{13}C-labeling since a C1-unit (methyl-THF) is incorporated during its biosynthesis from (naturally labeled) aspartate. As predicted, the C1S-Aux + pZ-BKACE15-BPT strain showed protein-derived serine and methionine pools consisting almost exclusively of single-labeled (M+1) molecules (>95 %). Supplementation of unlabeled formate (5 mM) in the medium, in addition to ^{13}C-β-alanine, abolished labeling in serine and methionine, demonstrating that excess exogenous formate "outdilutes" the intracellularly formed ^{13}C-formate. As a negative control, we included *E. coli* SIJ488 Δ*rutE* Δ*ydfG* + pZ-BKACE15-BPT, which lacks the auxotrophy of the C1S-Aux strain and its chromosomally integrated genes (CORE cycle module 4). This control strain showed exclusively unlabeled serine and methionine, confirming that incorporation of atoms from ^{13}C-β-alanine into central metabolism is negligible unless formate is assimilated through module 4 of the CORE cycle. Bar graphs represents mean values of three independently grown cultures. Source data are provided as a Source Data file.

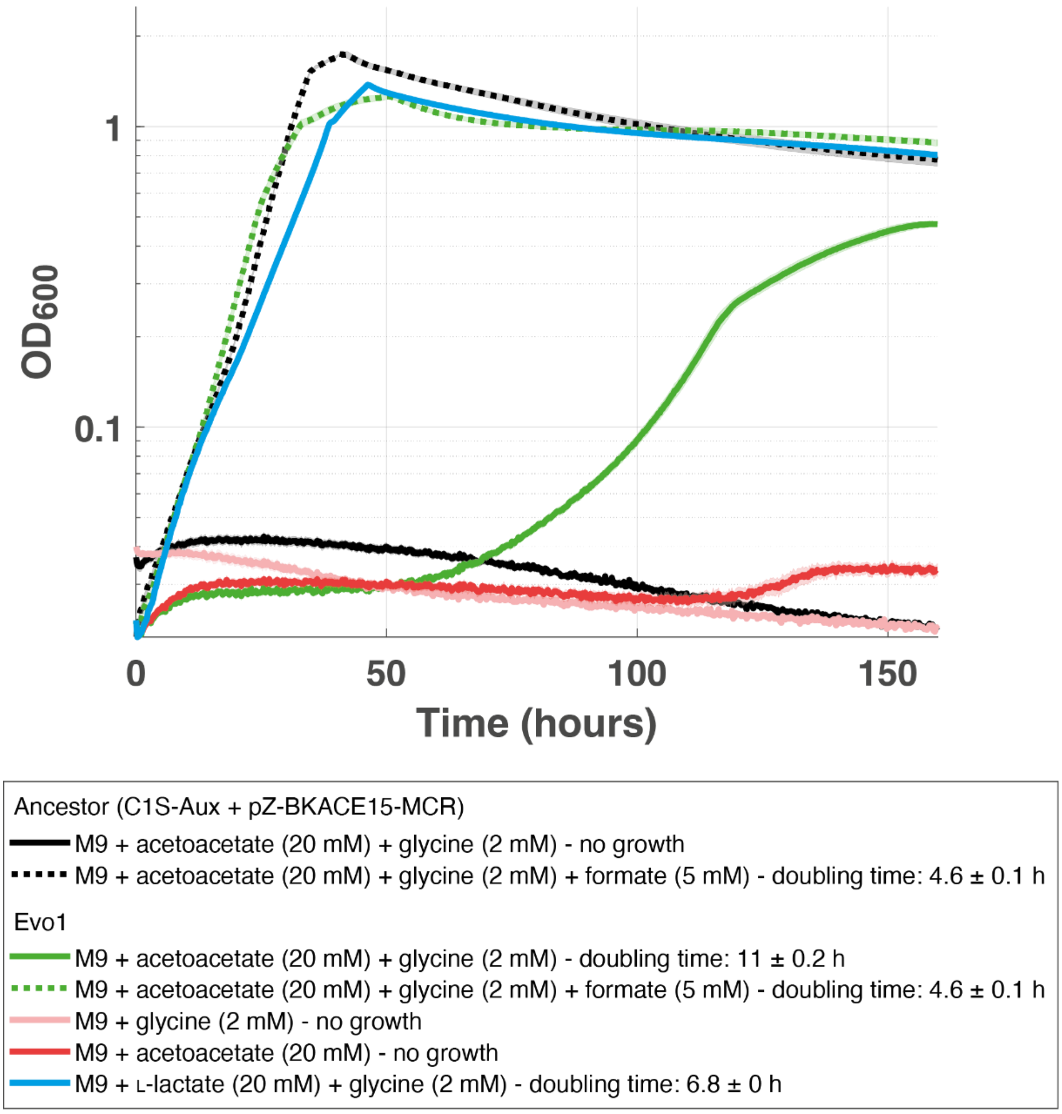

Supplementary Fig. 20. Growth characterization of Evo1. Shown growth curves represent the mean of technical triplicates, cultivated in a 96-well plate. Six technical replicates were used for the acetoacetate medium (green and black solid lines). Shaded areas surrounding the curve represent the standard deviation. Media lacking either acetoacetate or glycine, respectively, were used as negative controls (red lines). Media with added formate (5 mM) was used as a positive control (dashed lines). Growth on L-Lactate, requiring only CORE cycle modules 1,2,4, but not module 3 is shown for comparison (blue line). Source data are provided as a Source Data file.

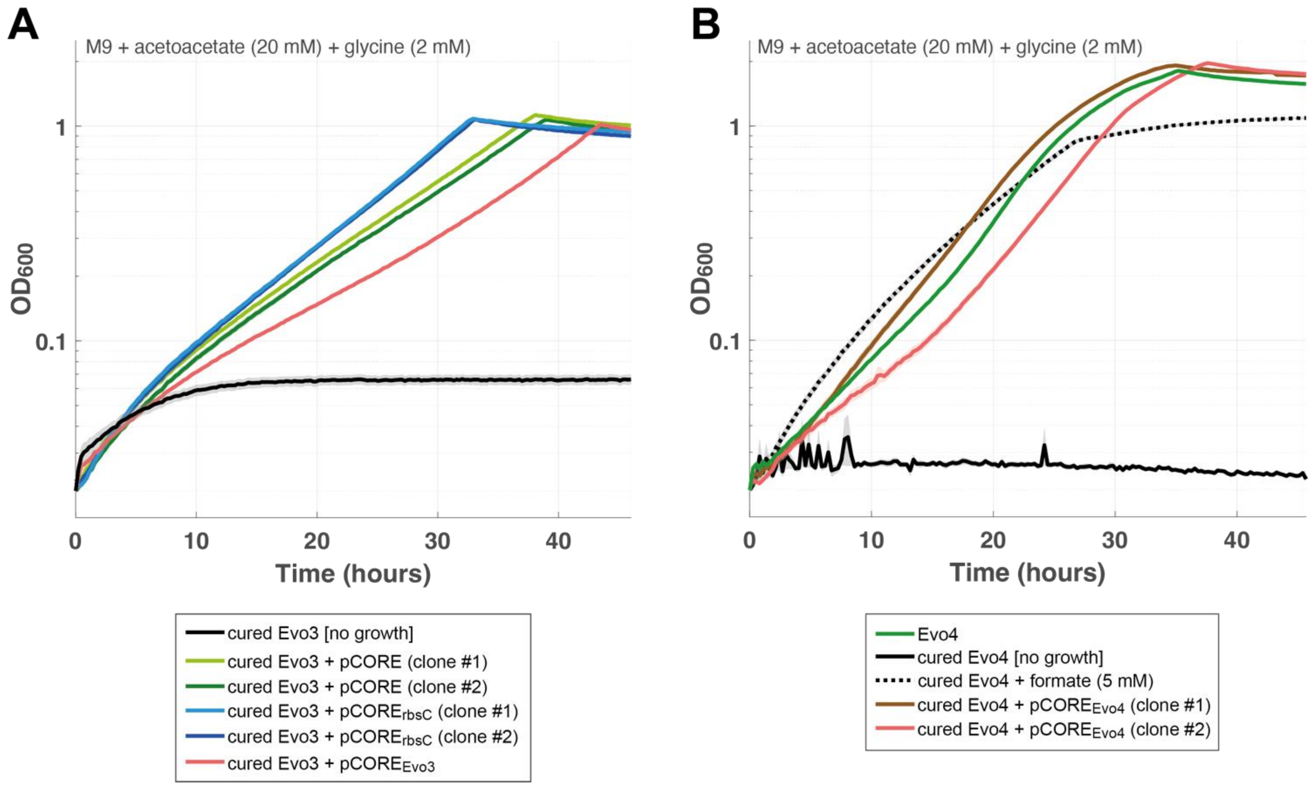

Supplementary Fig. 21. In-depth characterization of Evo3 and Evo4. Growth characterization of cured Evo3 and Evo4, re-transformed with different variants of pCORE. **A)** Removal of the plasmid from Evo3 abolishes CORE cycle-dependent growth on M9 + acetoacetate (20 mM) + glycine (2 mM) ("cured Evo3", black line; no growth above OD 0.1). Green lines show the growth of two biological replicates of cured Evo3 re-transformed with the non-evolved plasmid (pCORE). Blue lines show the growth of two biological replicates of cured Evo3 re-transformed with a plasmid variant bearing a weaker RBS for translation of the MCR gene ($pCORE_{rbsC}$). The red line shows growth of the cured Evo3 strain which was re-transformed with its own evolved plasmid (plasmid isolated from Evo3; "$pCORE_{Evo3}$"). Viability of the cured, plasmid-free strain was further ensured by the preculture in CORE cycle-independent minimal medium supplemented with formate (M9 + acetoacetate (20 mM) + glycine (2 mM) + formate (5 mM); not shown). **B)** Growth of cured Evo4 on acetoacetate medium (M9 + acetoacetate (20 mM) + glycine (2 mM)) with 5 mM formate (dashed black line) and without formate (solid black line), compared to Evo4 prior to curing (green line). Re-transformation of cured Evo4 with its own evolved plasmid (isolated from Evo4; "pZ-BKACE15-MCR_{Evo4}") restored growth (red and brown lines, 2 biological replicates). All growth curves in (A) and (B) represent the mean of technical triplicates, cultivated in a 96-well plate at 37 °C. Shaded areas surrounding the curve represent the standard deviation. Source data are provided as a Source Data file.

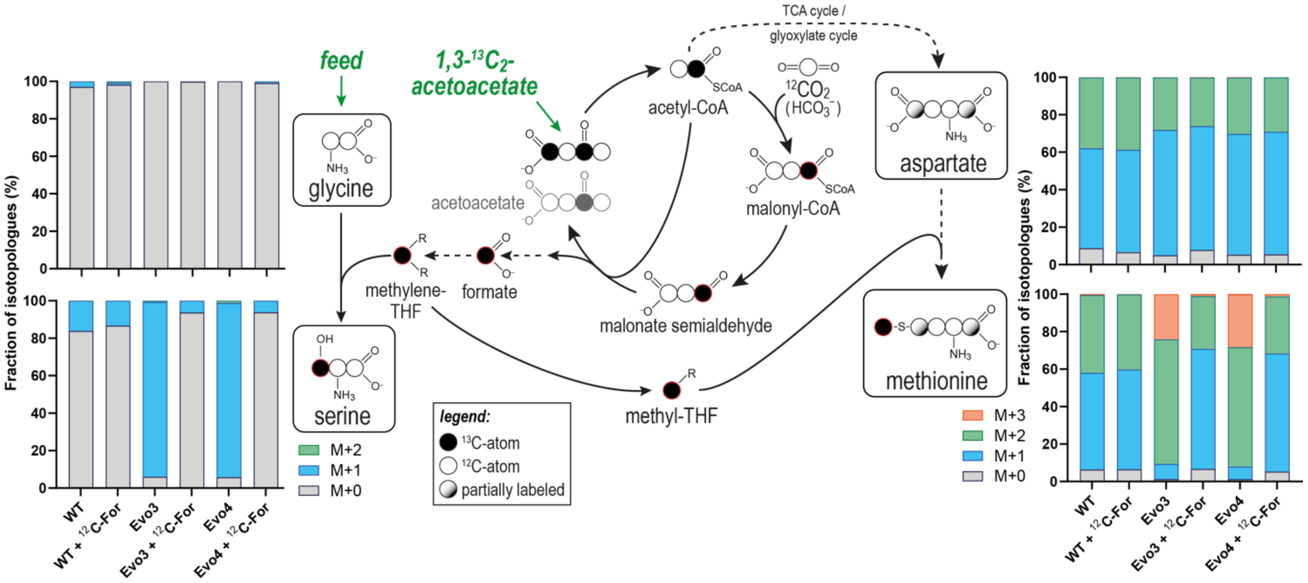

Supplementary Fig. 22. ^{13}C-tracing confirms operation of the complete CORE cycle (modules 1, 2, 3 and 4) in growing *E. coli* selection strains. Shown is the predicted and observed labeling pattern (isotopologue distribution) for selected protein-derived amino acids. The fastest evolved clones (Evo3 and Evo4) were cultivated in minimal medium containing positionally labeled 1,3-$^{13}C_2$-acetoacetate (M9 + 20 mM 1,3-$^{13}C_2$-acetoacetate + 2 mM glycine [unlabeled/naturally labeled]). For comparison, a wild-type *E. coli* strain (SIJ488) was cultivated in the same conditions to determine the native distribution of ^{13}C-labeling given the incorporation of labeled acetoacetate. Only when the complete CORE cycle is active and acts as the sole source of one-carbon units (C1-THF), serine is predicted to be single-labeled (its hydroxyl-carbon being derived from formate). Addition of exogenous unlabeled formate ("^{12}C-For", 5 mM) to the medium was used as a negative control, outcompeting intracellularly produced formate derived from the CORE cycle. Methionine biosynthesis represents addition of C1-THF (methyl-THF) to a carbon backbone derived from aspartate. ^{13}C-labeling in methionine is thus expected to represent the sum of any labeling present in aspartate (derived from acetoacetate via the TCA-cycle and glyoxylate cycle), plus one ^{13}C-atom derived from methyl-THF. Black circles indicate ^{13}C-atoms, white circles indicate ^{12}C-atoms, and shaded circles indicate atoms which can be either labeled or unlabeled in the mixed, cellular pool of the respective amino acid. Additionally, the atom representing the origin of formate (based on BKACE mechanism) is circled in red. Bar graphs represents mean values of three independently grown cultures. Source data are provided as a Source Data file.

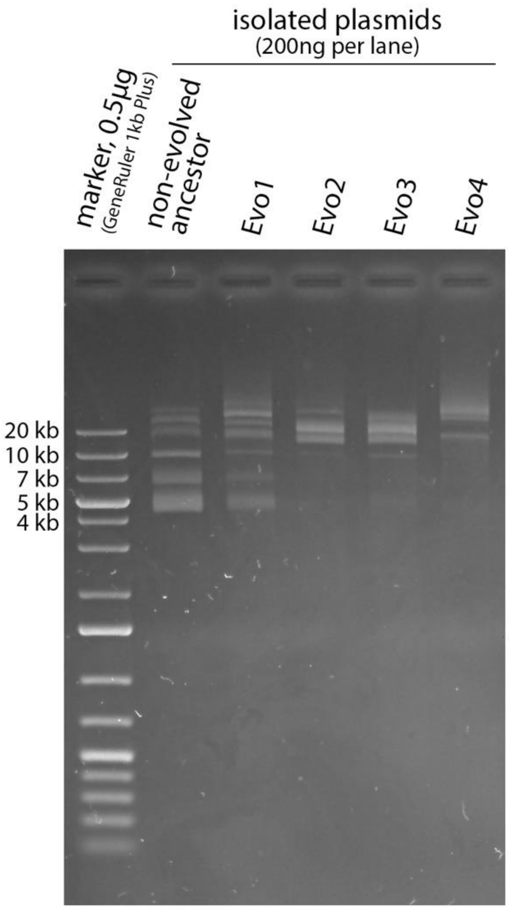

Supplementary Fig. 23. Detection of pCORE plasmid concatemers in evolved clones. Agarose gel electrophoresis of evolved plasmids to analyze their molecular weight compared to the plasmid isolated from the non-evolved ancestor (pCORE, expected size: 5.1kb). 200 ng of purified plasmid DNA were loaded per lane on a gel consisting of 1 % agarose in 1x TAE buffer. All plasmids for this analysis were isolated directly from the indicated strains (MG1655-derived), not from DH5a or similar *recA*- strains. Source data are provided as Source Data.

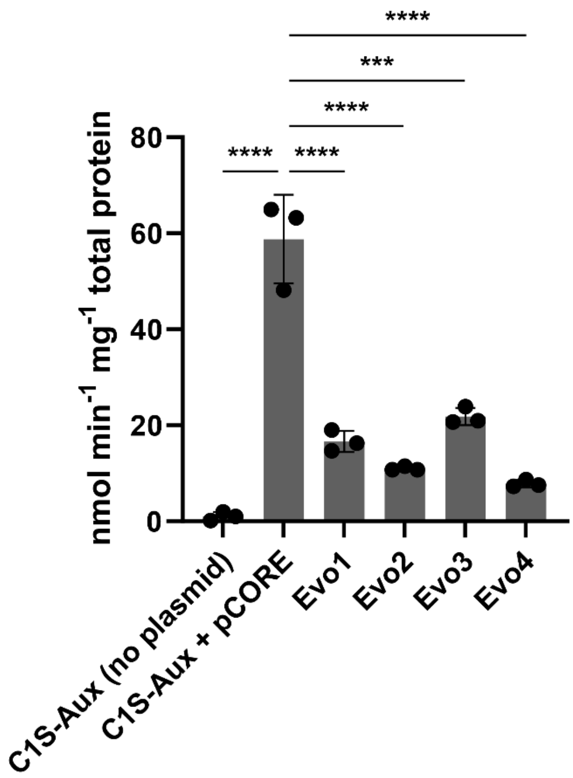

Supplementary Fig. 24. Cell-free activity assays of MCR. Activity of malonyl-CoA reductase (MCR) determined in cell-free extracts of the evolved strains, their non-evolved ancestor (C1S-Aux + pCORE) and the untransformed C1S-Aux strain serving as a negative control. Bars represent mean values derived from technical triplicates, with error bars indicating the standard deviation. For statistical analysis, Dunnett's multiple comparisons test was performed (GraphPad Prism), with adjusted p-values: *** $p = 0.0002$, **** $p < 0.0001$. Source data are provided as a Source Data file.

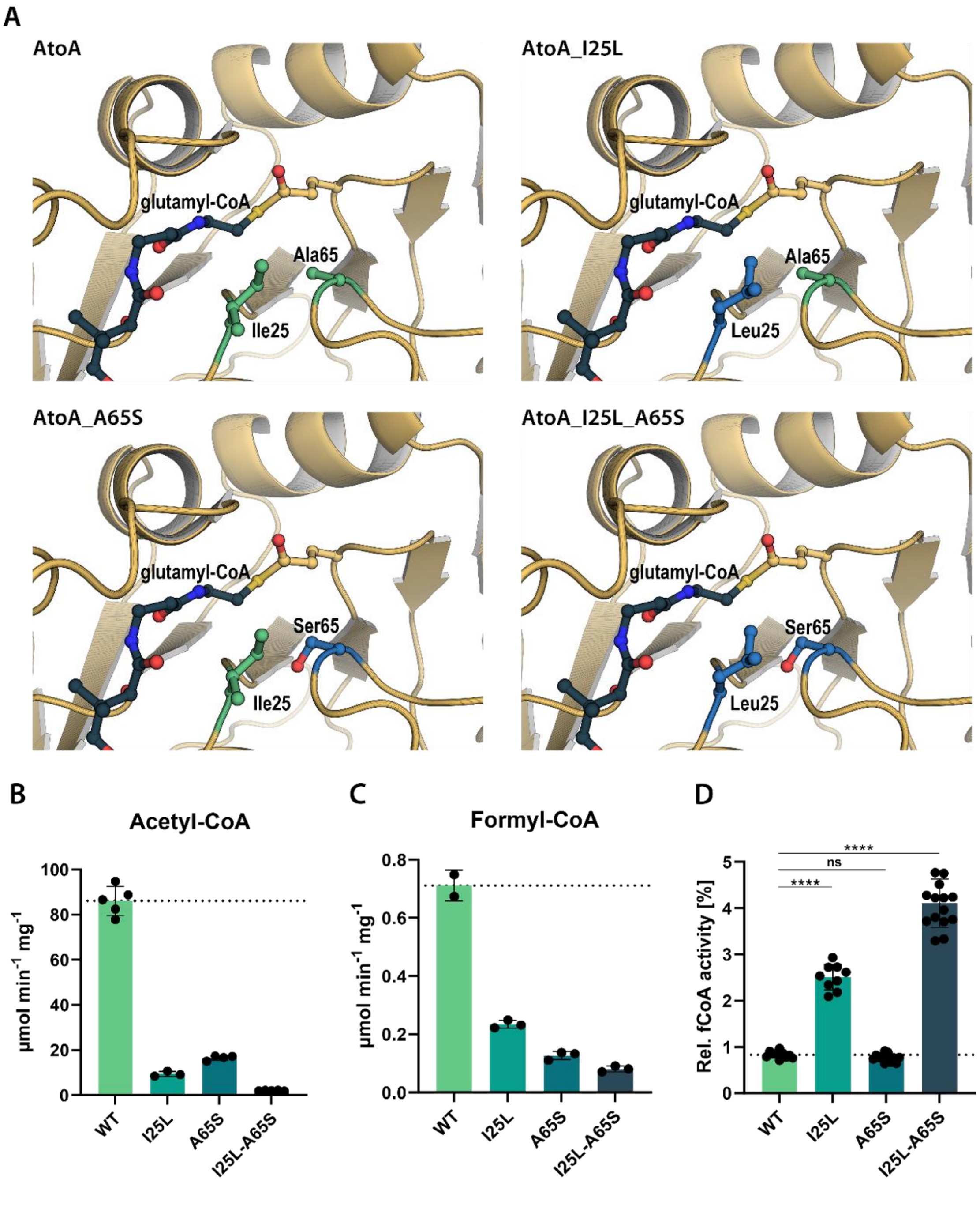

Supplementary Fig. 25. Homology models and kinetics of AtoA with I25L and A65S mutations. **A)** Homology modelling of AtoA_I25L based on PDB 3OXO with CoA covalently bound to an active site glutamate revealed that the mutated residue sits in the active site facing the acyl-moiety of CoA. Therefore, it is possible that the mutation influences the substrate binding. Homology modeling of AtoA_A65S showed that this mutation also sits in the direct proximity to the acyl-moiety of CoA in the active site and reduces the active site space impeding binding of bigger substrates (e.g. endogenous *E. coli* CoA-esters such as acetyl-CoA or succinyl-CoA). Therefore, this mutation might also have an impact on substrate binding. Homology modelling was done using SWISS-Model (see methods). [21] **B-C)** Spectrophotometric measurements of Acetyl-CoA:Acetoacetate-CoA transferase AtoDA with 1 mM acetyl-CoA **(B)** or 1 mM formyl-CoA **(C)**, 10 mM acetoacetate and 3-hydroxybutyryl-CoA dehydrogenase (PhaB) as a coupling enzyme. Mean values for n=2-5 technical replicates and individual data points are shown, error bars indicate standard deviation. **D)** Relative formyl-CoA activity was calculated by normalizing the spec. activity with formyl-CoA to the spec. activity with acetyl-CoA. Individual data points represent all combinatorial ratios of formyl-CoA and acetyl-CoA activities. For significance analysis, an unpaired *t*-test was performed. **** $p < 0.0001$, ns: non-significant. Source data are provided as a Source Data file.

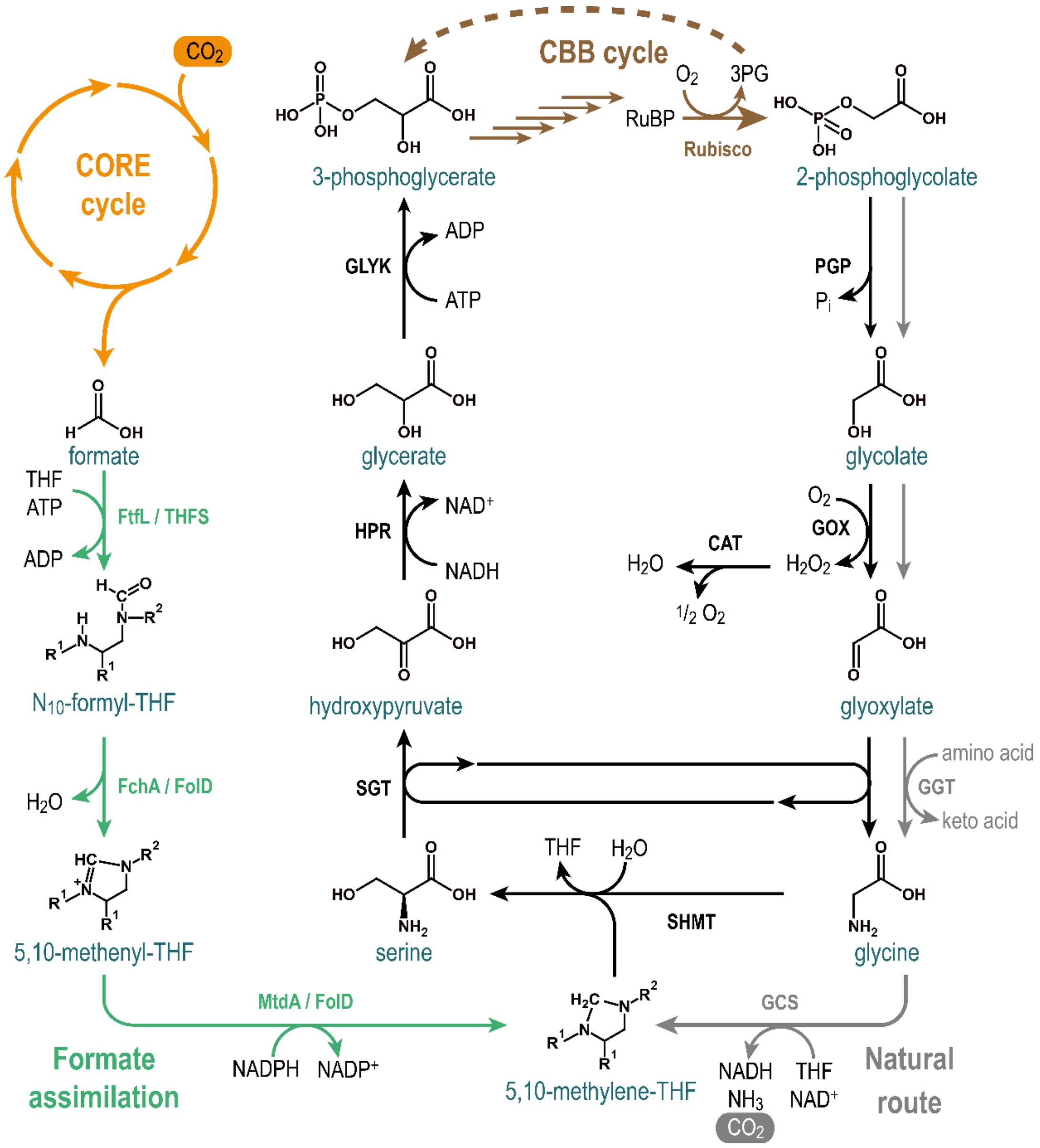

Supplementary Fig. 26. The CORE cycle as a photorespiratory bypass. Proposed synthetic photorespiration based on a CO_2 reduction pathway, circumventing the inefficient carbon-releasing reactions of natural photorespiration (glycine decarboxylation). The brown route highlights the Calvin-Benson-Bassham (CBB) cycle, the black and grey routes highlight the natural route of photorespiration in plants. The reactions shown in grey (indicating conversion of a second 2PG molecule in native photorespiration) are proposed to be replaced by the CORE cycle that produces formate (orange route), combined with its further conversion into the C1-carrier 5,10-methylene-THF (green route). In total, this bypass circumvents the release of CO_2 and ammonia, instead fixing an additional CO_2 molecule. CAT: catalase, FchA: 5,10-methenyl-THF cyclohydrolase, FolD: bifunctional 5,10-methenyl-THF cyclohydrolase/5,10-methylene-THF dehydrogenase, FtfL/THFS: formate-THF-ligase ("10-Formyl-THF synthetase"), GCS: glycine cleavage system, GGT: glutamate:glyoxylate aminotransferase, GLYK: glycerate 3-kinase, GOX: glycolate oxidase, HPR: hydroxypyruvate reductase, MtdA: 5,10-methylene-THF dehydrogenase, 3PG: 3-phosphoglycerate, PGP: 2-phosphoglycolate phosphatase, RuBP: ribulose-1,5-bisphosphate, SGT: serine:glyoxylate aminotransferase, SHMT: serine hydroxymethyltransferase, THF: tetrahydrofolate.

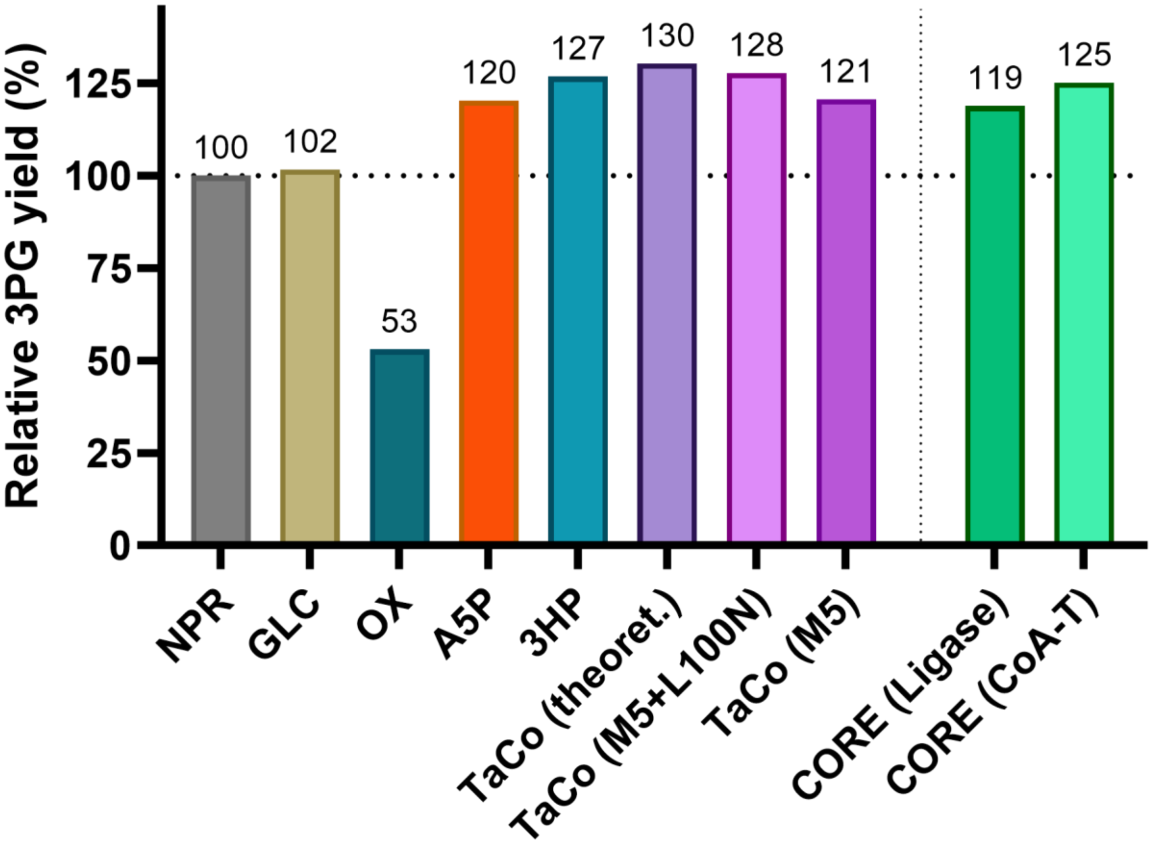

Supplementary Fig. 27. Efficiency of CORE-cycle based photorespiratory bypasses compared to native plant photorespiration and previously proposed bypasses. For a detailed view of the proposed photorespiratory bypass (i.e., integration of the CORE cycle into plant phosphoglycolate salvage), see Supplementary Fig. 24. Relative theoretical yields were calculated by comparing the amount of NADPH and ATP required to produce 3-phosphoglycerate from three molecules of CO_2 (see Methods, Supplementary Table 8, Supplementary Data 5). Oxygenation was assumed to occur in 25 % of RuBisCO reactions [22,23]. Source data are provided as a Source Data file.

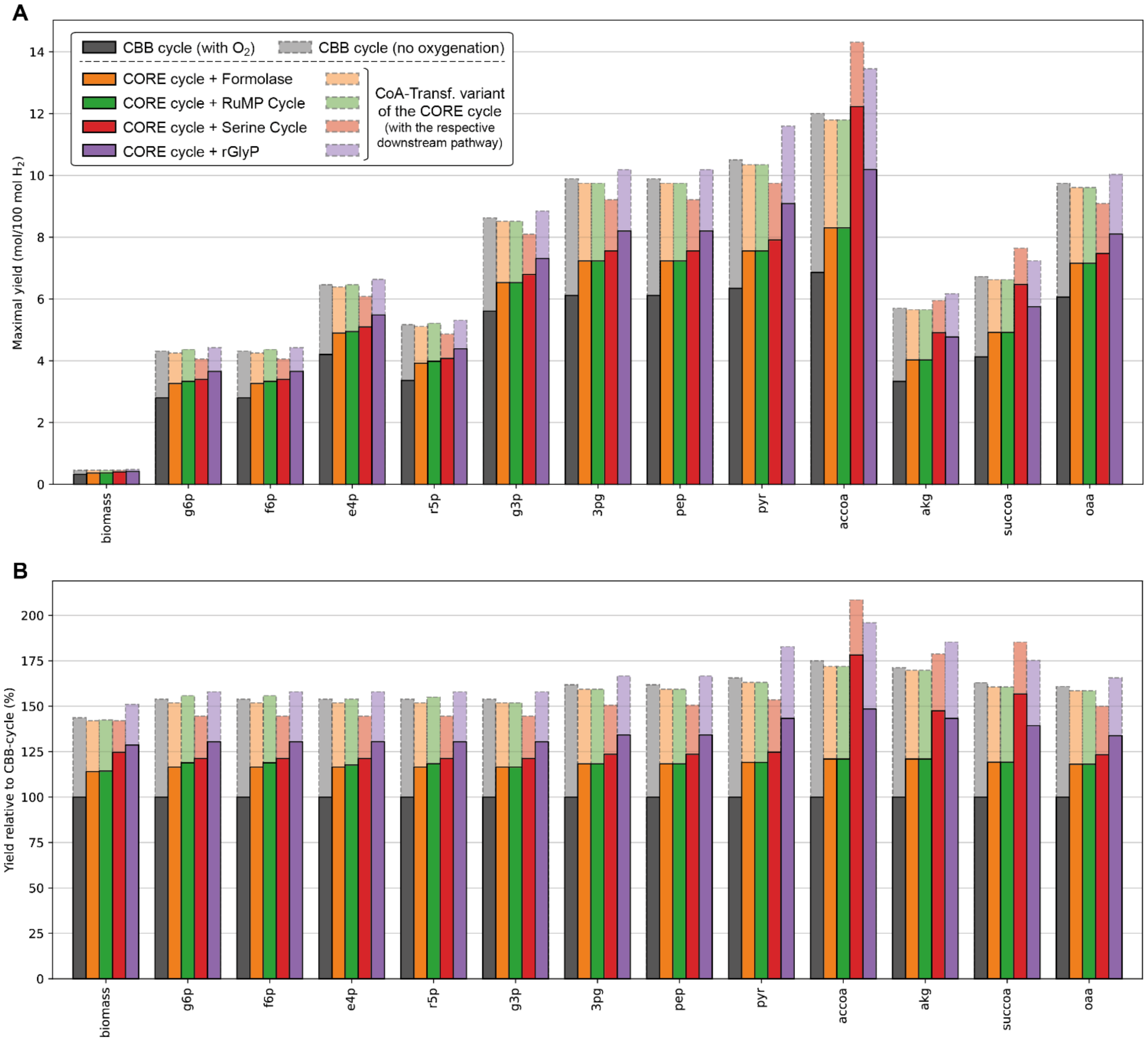

Supplementary Fig. 28. Stoichiometric modeling of synthetic autotrophy via the CORE cycle. Estimated yields of biomass and 12 individual bioproduction/biomass precursors for growth on H_2 and CO_2 alone, i.e. using the CORE cycle for autotrophic growth. An adapted *E. coli* genome-scale model was used for calculation of maximal theoretical product yield *via* flux balance analysis (see Methods). Grey bars represent the CBB cycle as a benchmark for comparison (dark grey solid bar: assuming oxygenation occurring in 25% of all RuBisCO reactions, thus requiring photorespiration via the bacterial glycerate pathway [24,25]; light grey dashed bars: CBB cycle without oxygenation, i.e. assuming growth in elevated CO_2 concentrations or with carbon-concentrating mechanisms). All other bars represent synthetic pathways based on the CORE cycle combined with different downstream formate assimilation routes. For the formolase pathway and RuMP cycle, formate reduction to formaldehyde [26] was integrated (formate + ATP + NADPH → formaldehyde + ADP + P_i + $NADP^+$). Solid bars show results for the "ligase/synthetase variant" of the CORE cycle which invests 3 ATP per reduction of CO_2 to formate (see main text and Supplementary Fig. 1; CO_2 + 3 ATP + NADPH → formate + 3 ADP + P_i + $NADP^+$). Dashed, transparent bars represent yields of the more ATP-efficient "CoA-transferase variant" of the CORE cycle (investing only 1 ATP per CO_2-reduction; Supplementary Fig. 1). **A)** Absolute molar yields for each considered product (mol product per 100 mol H_2). **B)** Relative yields of different routes, normalized to the CBB cycle (with oxygenation). **Abbreviations:** CBB = Calvin-Benson-Bassham; CoA-Transf. = CoA-transferase; rGlyP = reductive glycine pathway; RuMP = ribulose monophosphate cycle (with formate reduction to formaldehyde); bioproduction precursors (from left to right): g6p = glucose-6-phosphate, f6p = fructose-6-phosphate, e4p = erythrose-4-phosphate, r5p = ribose-5-phosphate, g3p = glyceraldehyde-3-phosphate, 3pg = 3-phosphoglycerate, pep = phosphoenolpyruvate, pyr = pyruvate, accoa = acetyl-CoA, akg = α-ketoglutarate, succoa = succinyl-CoA, oaa = oxaloacetate. Source data are provided as a Source Data file.

Supplementary Table 1. Comparison of candidate pathways for aerobic CO_2 reduction to formate.

Supplementary Figure #	Pathway name	ATP cost per formate	Total # of enzymes	# of new-to-nature enzymes	Reducing eq.
1	BKACE – Synthetase Variant	3	5 (6[a])	1 (2[a])	1 NADPH
	BKACE – Kinase Variant	2	6-7 (8[a])	1 (2[a])	1 NADPH
	BKACE – CoA-Transferase Variant	1 (2[b])	5 (6[b])	2	1 NADPH
2	Reductive Formamide	3	3	1	1 NAD(P)H
	Reductive Formamide– CoA Variant	3	4	2	1 NAD(P)H
3	Oxaloacetate Hydrolase – Synthetase Variant	3	4	1	1 NADPH
	Oxaloacetate Hydrolase – Kinase Variant	2	5	1	1 NADPH
4	Aspartate-Formate-Lyase – PC variant	1 (2[c])	4 (5[c])	1 (published)	1 NADPH (Fd[c])
	Aspartate-Formate-Lyase – PEPC variant	2 (3[c])	5 (6[c])	1 (published)	1 NADPH (Fd[c])
5	Thiolase – Synthetase Variant	3	4	2 (3[a])	1 NADPH
	Thiolase – CoA-Transferase Variant	1	4 (5[a])	2 (3[b])	1 NADPH
6	Amino Acid Thiolase	2	6 (7[a])	1 (2[a])	1 NADPH
7	Formyl-Pyruvate Hydrolase	2	6	2	1 NADPH
8	HACL-TaCo – Synthetase variant	5	6 (7[a])	5 (3 established)	1 NADPH
	HACL-TaCo – CoA-Transferase variant	3 (4[b])	6 (7[b])	5 (3 established)	1 NADPH
9	3HB-Acetone – Synthetase variant	4	5 (6[a])	0 (1[a])	1 NAD(P)H
	3HB-Acetone – CoA-Transferase variant(s)	2 (3[b])	5	1	1 NAD(P)H
10	3HP-Acetyl-CoA – Synthetase variant	4	5 (6[a])	2 (3[a]) (1 established)	1 NAD(P)H
	3HP- Acetyl-CoA – CoA-Transferase variant(s)	2 (3[b])	5	3	1 NAD(P)H

[a] if a novel thioesterase is required for sufficiently fast formyl-CoA hydrolysis (instead of spontaneous hydrolysis or a natural CoA-transferase)
[b] accounting for undesired spontaneous formyl-CoA hydrolysis and thus the requirement for an additional “backup” donor/enzyme for CoA-transfer
[c] in plants (glutamate biosynthesis via the ATP-dependent chloroplastic glutamate synthase cycle with ferredoxin as reductant); Fd = ferredoxin

Supplementary Table 2. Kinetic properties of CORE cycle enzymes.

Enzyme	Full name	Organism	Spec. activity [μmol min^{-1} mg^{-1}] [a]	k_{obs} [s^{-1}] [a]	Uniprot ID	Reference
Acl_Sl	Acetoacetate CoA Ligase	*Streptomyces lividans*	63 ± 7	79 ± 9	D6EQU8	this work
Acl_Sz	Acetoacetate CoA Ligase	*Shinella zoogloeoides* (*Zoogloea ramigera*) [b]	117 ± 2	144 ± 3	- [b]	this work
AtoDA	Acetyl-CoA:Acetoacetate CoA Transferase	*Escherichia coli*	86.1 ± 6.5	68.3 ± 5.2	P76458 & P76459	this work
AtoB	Acetyl-CoA Acetyltransferase	*Escherichia coli*	289	202	P76461	this work
Pcc_Me_D407I	Propionyl-CoA Carboxylase (mutant D407I)	*Methylobacterium extorquens*	1.15	2.5	C5AWU5 & C5AP75 [c]	this work
MCR	Malonyl-CoA Reductase (C-term. domain)	*Chloroflexus aurantiacus*	9.5 ± 0.3	9.5 ± 0.3	Q6QQP7 [d]	this work
Mcr_St	Malonyl-CoA Reductase	*Sulfolobus tokodaii*	2.1 ± 0.1	1.4 ± 0.1	Q96YK1	this work

[a] Spec. activities and k_{obs} were measured at substrate-saturated conditions and should approximate V_{max} and k_{cat} (see method section for details).
[b] There is no corresponding Uniprot entry. The sequence was obtained from [27] (*Zoogloea ramigera* has been reclassified to *Shinella zoogloeoides*).
[c] We used a mutant variant of this protein.
[d] We used a dissected version of this bifunctional protein (using only the C-terminal domain, comprising the residues S550 to V1219 of UniProt Q6QQP7) [28].

Supplementary Table 3. Specific activities for the top 20 BKACE candidates from the high-throughput screen.

Candidate	Organism	Uniprot ID	Structural group	Spec. activity [nmol min^{-1} mg^{-1}]
BKACE137	*Acidiphilium cryptum*	A5G287	G1	54.04
BKACE15	*Paracoccus denitrificans*	A1B802	G1	46.50
BKACE407	*Cupriavidus metallidurans*	Q1LCH6	G1	39.52
BKACE314	*Paraburkholderia phytofirmans*	B2T139	G1	28.21
BKACE298	*Burkholderia ambifaria*	B1Z3A7	G1	27.17
BKACE244	*Bordetella petrii*	A9I628	G1	25.97
BKACE393	*Roseobacter denitrificans*	Q160J5	G1	23.97
BKACE179	*Ochrobactrum anthropi*	A6X326	G5	19.34
BKACE178	*Ochrobactrum anthropi*	A6X2V8	G1	16.47
BKACE227	*Sulfitobacter indolifex*	A9DSW8	G5	12.30
BKACE242	*Roseobacter litoralis*	A9HSN9	G5	11.33
BKACE379	*Cupriavidus necator*	Q0KFN7	G1	9.04
BKACE274	*Paraburkholderia graminis*	B1G5Y9	G1	8.18
BKACE10	*Mycolicibacterium smegmatis*	A0R5Z8	G3	7.05
BKACE4	*Labrenzia aggregata*	A0NVX0	G5	7.04
BKACE82	*Roseovarius nubinhibens*	A3SRB7	G5	6.75
BKACE476	*Pseudomonas syringae pv. Syringae*	Q4ZUE4	G1	5.39
BKACE236	*Phaeobacter gallaeciensis*	A9GCI6	G5	4.93
BKACE479	*Ruegeria pomeroyi*	Q5LPZ3	G5	1.85
BKACE395	*Roseobacter denitrificans*	Q166L4	G5	1.15

Activities were determined in presence of 250 µM acetyl-CoA and 1.2 mM MSA in 50 mM Tris-HCl (pH 7.5) by LC-MS/MS analysis. All measurements were done in duplicates, mean values are given for specific activities.

Supplementary Table 4. Kinetic properties of selected BKACE homologs.

Enzyme	Organism	Uniprot ID	Spec. activity [nmol min^{-1} mg^{-1}]	Turnover number [min^{-1}]
BKACE15	*Paracoccus denitrificans*	A1B802	761 ± 20	27.7 ± 0.8
BKACE244	*Bordetella petrii*	A9I628	324 ± 10	12.1 ± 0.4
BKACE298	*Burkholderia ambifaria*	B1Z3A7	298 ± 41	10.3 ± 1.5
BKACE407	*Cupriavidus metallidurans*	Q1LCH6	273 ± 24	9.5 ± 0.9
BKACE393	*Roseobacter denitrificans*	Q160J5	196 ± 12	6.8 ± 0.5
BKACE10	*Mycolicibacterium smegmatis*	A0R5Z8	189 ± 7	6.5 ± 0.3
BKACE314	*Paraburkholderia phytofirmans*	B2T139	168 ± 14	5.9 ± 0.5
BKACE137	*Acidiphilium cryptum*	A5G287	66 ± 7	2.3 ± 0.3
BKACE15_E171D	*Paracoccus denitrificans*	-	40 ± 1	1.5 ± 0.1
BKACE15_E171L	*Paracoccus denitrificans*	-	n.d.	n.d.

Homologs were chosen from top 20 BKACE candidates in the initial high throughput screen. Mutant variants were developed based on BKACE15. Reaction kinetics were determined using 4 mM MSA and 1 mM acetyl-CoA. All measurements were done in triplicates. Quantification of acetoacetate formation over time was used to determine enzyme activities with standard deviations. n.d. = non-detectable.

Supplementary Table 5. Michaelis-Menten kinetics of BKACE15.

	**Acetyl-CoA**	**MSA**
V_{max}	745 ± 15 nmol min^{-1} mg^{-1}	768 ± 26 nmol min^{-1} mg^{-1}
k_{cat}	27.1 ± 0.6 min^{-1}	27.9 ± 1.0 min^{-1}
K_M	0.11 ± 0.01 mM	0.83 ± 0.09 mM
k_{cat}/K_M	4.22 x 10^3 M^{-1} s^{-1}	5.63 x 10^2 M^{-1} s^{-1}

Measurements were done with 5 mM MSA and varying concentrations of acetyl-CoA, or 2 mM acetyl-CoA and varying concentrations of MSA. All measurements were done in triplicates. Quantification of acetoacetate formation over time was used to determine enzyme activities with standard deviations.

Supplementary Table 6. Mutations identified in evolved clones.

Gene	Description	Evo1	Evo2	Evo3	Evo4
partially mutated: mcr (Y331)*	malonyl-CoA reductase	76.9 %	69.5 %	56.3 %	74.9 %
atoA (I25L)	acetyl-CoA:acetoacetyl-CoA transferase subunit beta	X	X	X	X
atoE (C422F)	short chain fatty acid transporter	X	X	X	X
atoE (M158I)	short chain fatty acid transporter		X	X	X
Δ112kb (IS2-mediated)	97 genes Δ(ydbA-ydeM), including *aldA, patD, adhP, fdnGHI, maeA, gadB*		X	X	X
dksA (A66T)	RNA polymerase-binding transcription factor			X	
hupB (Δ9bp)	DNA-binding protein HU-beta			X	
Iap/cas2 (intergenic transposon insertion)	alkaline phosphatase isozyme conversion protein/CRISPR-associated endoribonuclease Cas2			X	
nth / dtpA (intergenic Δ1bp)	endonuclease III/dipeptide/tripeptide:H(+) symporter DtpA			X	
mutT	Transposon insertion (IS)				X
75 additional mutations (Supplementary Data 1)					X

Shown are only mutations gained during evolution, i.e. not yet present in the non-evolved ancestor "C1S-Aux + pCORE" (see Supplementary Data 1 for complete list of sequenced clones and all identified mutations). An "X" indicates that the respective mutation is present in the clone. Percentages indicate frequency of the mutated allele, as reported in the "marginal predictions" by breseq [29].

Supplementary Table 7. Kinetic properties of Acetyl-CoA:Acetoacetate-CoA transferase AtoDA.

Enzyme	ALE lineage	Spec. activity [µmol min^{-1} mg^{-1}]	Turnover number [s^{-1}]	Spec. activity [µmol min^{-1} mg^{-1}]	Turnover number [s^{-1}]	Relative formyl-CoA activity [%]
		Acetyl-CoA		Formyl-CoA		
AtoDA	wild type	86.1 ± 6.5	68.3 ± 5.2	0.71 ± 0.06	0.56 ± 0.05	0.8 ± 0.1
AtoDA_I25L	Evo1, Evo2, Evo3, Evo4	9.4 ± 1.1	7.5 ± 0.9	0.23 ± 0.02	0.19 ± 0.01	2.5 ± 0.3
AtoDA_A65S	Evo1a	16.6 ± 1.1	13.1 ± 0.9	0.13 ± 0.02	0.10 ± 0.01	0.8 ± 0.1
AtoDA_I25L-A65S	-	2.0 ± 0.2	1.6 ± 0.2	0.08 ± 0.01	0.06 ± 0.08	4.1 ± 0.53

CoA transferase activities were measured with 1 mM acetyl-CoA / formyl-CoA, 10 mM acetoacetate and 3-hydroxybutyryl-CoA dehydrogenase (PhaB) as a coupling enzyme. Relative formyl-CoA activity was calculated by normalizing the spec. activity with formyl-CoA to the spec. activity with acetyl-CoA.

Supplementary Table 8. Comparison of natural photorespiration and synthetic photorespiration bypasses, adapted from [11].

	NPR	GLC [30]	OX [31,32]	A5P [10]	3HP [33]	TaCo pathway (theoretical) [10,11]	TaCo pathway (M5) [11]	TaCo pathway (M5+L100N) [34]	CORE cycle (CoA-T. variant)	CORE cycle (Ligase variant)
CO_2 release	yes	yes	yes	no	no	no	no	no	no	no
Place of CO_2 release	mitochondria	chloroplast (near RuBisCO)	chloroplast (near RuBisCO)	-	-	-	-	-	-	-
Carbon efficiency [a]	75%	75%	0%	100%	150%	150%	150%	150%	150%	150%
Route for (re-)fixation of released CO_2 [b]	complete CBB cycle	complete CBB cycle	complete CBB cycle	part of CBB cycle	Photorespiratory bypass fixes CO_2	Photorespiratory bypass fixes CO_2	Photorespiratory bypass fixes CO_2	Photorespiratory bypass fixes CO_2	Photorespiratory bypass fixes CO_2	Photorespiratory bypass fixes CO_2
NH_3 release	yes	no	no	no	no	no	no	no	no	no
Required enzymes (excluding CBB cycle)	11	5	6	5	12	6 [c]	6 [c]	6 [c]	8 (+ 8 from NPR)	8 (+ 8 from NPR)
**Required turns of the CBB cycle [b]**	**4.8**	**4.8**	**12**	**4**	**3**	**3**	**3**	**3**	**3**	**3**
**RuBisCO carboxylations (75%)**	**3.6**	**3.6**	**9**	**3**	**2.25**	**2.25**	**2.25**	**2.25**	**2.25**	**2.25**
**RuBisCO oxygenations (25%)**	**1.2**	**1.2**	**3**	**1**	**0.75**	**0.75**	**0.75**	**0.75**	**0.75**	**0.75**
**consumed ATP**	**14**	**13.4**	**32**	**12**	**11.75**	**11**	**13.2**	**11.5**	**10.25**	**11.75**
**consumed reducing equivalents [d]**	**8 NAD(P)H + 0.6 Fd^{2-}**	**8.6 NAD(P)H**	**14 NAD(P)H [d]**	**7 NAD(P)H**	**6.5 NAD(P)H**	**6.5 NAD(P)H**	**6.5 NAD(P)H**	**6.5 NAD(P)H**	**7.25 NAD(P)H**	**7.25 NAD(P)H**
**total consumed "ATP-equivalents" [e]**	**35.5**	**34.9**	**67**	**29.5**	**28**	**27.25**	**29.425**	**27.775**	**29.875**	**28.375**
**Energetic efficiency (relative to NPR)**	**100%**	**102%**	**53%**	**120%**	**127%**	**130%**	**121%**	**128%**	**119%**	**125%**

[a] for conversion of 2-phosphoglycolate into 3-phosphoglycerate

[b] we note that the CO_2-assimilating photorespiratory bypasses achieve some of their predicted energetic advantage (lower resource cost) based on the fact that they require fewer turnovers (and corresponding resource investments) of the CBB cycle for formation of the same molar amount of product (here: 1 molecule of 3-phosphoglycerate from 3 molecules of CO_2) due to additional carbon fixation occurring in the photorespiration bypass.

[c] for the TaCo pathway, we considered the two reactions of TCR (tartronyl-CoA reductase) as two enzymatic pathway steps

[d] we note that differences in total consumed reducing equivalents are due to differences in turnovers of oxygenation and glycolate oxidation (which transfer electrons to molecular oxygen); redox balance of the "OX" route is calculated assuming that glycolate oxidation transfers $2e^-$ to oxygen (not NAD^+), since the physiological electron acceptor (and energy conservation) of "glycolate dehydrogenase" is unknown [30]

[e] required "ATP-equivalents" were calculated based on the equations: 1 NAD(P)H = 2.5 ATP [35,36], 2 reduced ferredoxins (1 Fd^{2-}) = 1 NAD(P)H

bold numbers are the result of flux-balance analysis, calculated for net conversion of 3x CO_2 into one unit of 3-phosphoglycerate via the CBB cycle and the respective photorespiration route (see Supplementary Data 5 for all individual reactions and flux values; or see "Data availability" for deposited code)

Abbreviations: Fd^{2-} = 2 reduced ferredoxins; NPR = native plant photorespiration (glycine decarboxylation route); GLC: glycerate bypass, OX: glycolate oxidation pathway, A5P: arabinose-5-phosphate shunt, 3HP: 3-hydroxypropionate bypass; TaCo: tartronyl-CoA pathway; M5 = includes futile ATP-hydrolysis by the enzyme "GCC(M5)" in the TaCo pathway (3.9 ATP per tartronyl-CoA formed); M5+L100N = includes futile ATP-hydrolysis by the optimized enzyme "GCC(M5+L100N)" in the TaCo pathway (1.7 ATP per tartronyl-CoA formed); CoA-T = CoA-transferase variant (see Supplementary Fig. 1).

Supplementary Table 9. Parameter settings for LC-MS/MS-based quantification of CoA-esters.

Name	Precursor ion	Product ion	Collision energy [V]	Fragmentor voltage [V]	Cell accelerator voltage [V]	Dwell time [msec]	Polarity
CoA quantifier	768.12	428.1	31	130	5	150	Positive
CoA qualifier	769.12	261.1	31	130	5	150	Positive
Acetyl-CoA quantifier	810.1	428.1	35	130	5	150	Positive
Acetyl-CoA qualifier	810.1	302.2	35	130	5	150	Positive
Formyl-CoA quantifier	796.1	428.1	23	130	5	150	Positive
Formyl-CoA qualifier	796.1	289.4	31	130	5	150	Positive

Supplementary Table 10. Parameter settings for LC-MS/MS-based quantification of acetoacetate.

Name	Precursor Ion	Product Ion	Collision energy [V]	Fragmentor Voltage [V]	Cell Accelerator Voltage [V]	Dwell time [msec]	Polarity
Acetoacetate quantifier	101	57.1	8	80	5	400	Negative
Acetoacetate qualifier	101	101	0	80	5	400	Negative

Supplementary Table 11. X-ray data collection and structure refinement statistics.

**Accession**	**PDB 8RIO**	**PDB 8RIP**	**PDB 9HNF**
**Ligands**	**Zn^{2+}, L-proline**	**Zn^{2+}, malonate, CoA**	**Zn^{2+}, acetoacetate, acetyl-CoA**
**Data collection**			
Beamline	PETRA III - P14	PETRA III - P14	PETRA III - P13
Wavelength (Å)	0.9763	0.9763	0.9763
Space Group	$C\ 2\ 2\ 2_1$	$P\ 2_1\ 2_1\ 2_1$	$C\ 2\ 2\ 2_1$
Unit cell dimensions<br>a, b, c (Å)<br>α, β, γ (°)	81.07, 137.871, 132.52 90.00, 90.00, 90.00	79.16, 134.35, 140.85 90.00, 90.00, 90.00	80.900, 138.480, 136.360 90.00, 90.00, 90.00
Resolution (Å)	24.78 - 1.45 (1.53 - 1.45)	29.99 - 1.81 (1.91 - 1.81)	27.69 - 2.10 (2.16 - 2.10)
Unique reflections	129609 (18038)	136868 (19659)	44681 (3628)
Multiplicity	12.1 (10.8)	9.1 (9.2)	13.2 (13.5)
Completeness (%)	99.1 (95.5)	99.8 (99.2)	99.4 (99.5)
$I / \sigma I$	19.2 (5.9)	18.4 (2.3)	14.4 (3.2)
R_{merge}	0.090 (0.499)	0.069 (0.908)	0.1114 (0.841)
R_{pim}	0.026 (0.157)	0.024 (0.313)	0.032 (0.234)
$CC_{1/2}$	0.997 (0.929)	0.999 (0.811)	0.998 (0.884)
**Refinement**			
R_{work} / R_{free}	0.1564 / 0.1768	0.1576/ 0.1780	0.1752 / 0.1920
RMS bonds	0.015	0.007	0.003
RMS angles	1.425	0.876	0.620
Ramachandran			
favored (%)	97.55	97.80	97.39
allowed (%)	2.45	2.20	2.61
outliers (%)	0.00	0.00	0.00
Rotamer outliers (%)	0.41	0.62	0.84
Number of atoms	5560	10467	5060
Protein	4720	9423	4698
Ligands	18	128	118
Solvent	822	916	253
Average B-factor	17.44	30.47	40.87
Protein	15.67	29.75	40.36
Ligands	18.24	38.26	57.36
Solvent	27.62	36.74	42.66

Values in parentheses correspond to the highest resolution shell.

Supplementary Table 12. Strains used in this study.

Strain abbreviation	Genotype	Purpose/Notes	Source
E. coli DH5α	F^- Φ80*lac*ZΔM15 Δ(*lac*ZYA-*arg*F) U169 *rec*A1 *end*A1 *hsd*R17(r_k^-, m_k^+) *pho*A *sup*E44 *thi*-1 *gyr*A96 *rel*A1 λ^-	Cloning and plasmid propagation	Thermo Fisher Scientific
E. coli NEB Turbo	F' $proA^+B^+$ $lacI^q$ ΔlacZM15 / fhuA2 Δ(lac-proAB) glnV galK16 galE15 R(zgb-210::Tn10)Tet^S endA1 thi-1 Δ(hsdS-mcrB)5	Cloning and plasmid propagation	NEB
E. coli BL21 DE3	fhuA2 [lon] ompT gal (λ DE3) [dcm] ΔhsdS λ *DE3* = λ sBamHIo ΔEcoRI-B int::(lacI::PlacUV5::T7 gene1) i21 Δnin5	Expression strain for protein purification	NEB
E. coli ArcticExpress DE3	*E. coli* B F^- *ompT hsdS*(r_B^- m_B^-) dcm^+ Tet^r *gal* λ(DE3) *endA* Hte [*cpn10 cpn60* $Gent^r$] *λ DE3* = λ sBamHIo ΔEcoRI-B int::(lacI::PlacUV5::T7 gene1) i21 Δnin5	Expression strain for protein purification of BKACE candidates	Agilent
ST18	*pro thi* $hsdR^+$ Tp^R Sm^R; chromosome::RP4-2 Tc::Mu-Kan::Tn7λpir Δ*hemA*	Conjugation donor for insertion of the chromosomal "module 4" (from pDM4:SS9-C_1M)	[37]
SIJ488	K-12 MG1655 Tn7::para-exo-beta-gam; prha-FLP; xylSpm-IsceI	Used as wild-type *E. coli*	[38]
-	SIJ488 Δ*frmRAB* Δ*serA* Δ*(gcvTHP)*	Strain auxotrophic to one-carbon units (C1-THF) and serine	[39]
-	SIJ488 Δ*frmRAB* Δ*serA* Δ*(gcvTHP)* *nhaR*::IS1::*oriT*-R6K(ori)-P_{pgi20}-rbsC_*ftfL*-rbsC_*fchA*-rbsC_*mtdA*::*rpsT*	above strain with a genomic insertion of CORE cycle module 4	This study
C1S-Y	SIJ488 Δ*frmRAB* Δ*serA* Δ*(gcvTHP)* Δ*ydfG::Km*^{R} *nhaR*::IS1::*oriT*-R6K(ori)-P_{pgi20}-rbsC_*ftfL*-rbsC_*fchA*-rbsC_*mtdA*::*rpsT*	as above, with additional deletion of *ydfG* (removed sink for malonate semialdehyde)	This study
C1S-Aux	SIJ488 Δ*frmRAB* Δ*serA* Δ*(gcvTHP)* Δ*rutE::Cm*^{R} Δ*ydfG::Km*^{R} *nhaR*::IS1::*oriT*-R6K(ori)-P_{pgi20}-rbsC_*ftfL*-rbsC_*fchA*-rbsC_*mtdA*::*rpsT*	as above, with additional deletion of *rutE* and *ydfG* (removed sinks for malonate semialdehyde)	This study
SIJ488 Δ*rutE* Δ*ydfG*	SIJ488 Δ*rutE::Cm*^{R} Δ*ydfG::Km*^{R}	Used as negative control strain for experiments with 13C-β-alanine	This study
Evo1, Evo1a-c	C1S-Aux + pCORE, see Supplementary Data 1 for mutations	Isolates from serial dilution	This study
Evo2, Evo2a-b, Evo3, Evo3a-d, Evo4, Evo4a-d	C1S-Aux + pCORE, see Supplementary Data 1 for mutations	Isolates from turbidostat	This study
Evo1 cured	Evo1, lacking the pCORE plasmid	Negative control	This study
Evo2 cured	Evo2, lacking the pCORE plasmid	Negative control	This study
Evo3 cured	Evo3, lacking the pCORE plasmid	Negative control	This study
Evo4 cured	Evo4, lacking the pCORE plasmid	Negative control	This study

Supplementary Table 13. Oligonucleotides used in this study.

Primer name	Sequence (5'-3')	Purpose
4945	acatcgcatcgcgaaaagtttgttgatcgacaatgttcatagtttgctccgtgtaggctggagctgcttc	amplification of CmR cassette from pKD3 for deletion of *rutE*
4946	ggcttgccagcctgttacatcaccgtgaagccgccctgtaaggaattgctcatatgaatatcctccttag	amplification of CmR cassette from pKD3 for deletion of *rutE*
4947	cggcgtatcggtcacactgc	Verification of *rutE* deletion
4948	gaacttcatgccgccctgcc	Verification of *rutE* deletion
1785_ydfG_KO_F	atcccgtgctatgttattgacacacaaaagcgttgaggaacagtgagatgattaaccctcactaaagggcg	amplification of KmR cassette from FRT-PGK-gb2-neo-FRT (GeneBridges) for deletion of *ydfG*
1786_ydfG_KO_R	taacccggcagttacgccgggtataaaaattactgacggtggacattcagtaatacgactcactatagggctc	amplification of KmR cassette from FRT-PGK-gb2-neo-FRT (GeneBridges) for deletion of *ydfG*
1787_ydfG_KO_V_F	agtaattctccgctttgttcc	Verification of *ydfG* deletion
1788_ydfG_KO_V_R	gtgacacattgaaacgaacag	Verification of *ydfG* deletion
P01	gttattgtctcatgagcggatac	PCR-verification and sequencing of pZASS-based plasmids, including pCORE
P02	ggcggatttgtcctactcag	PCR-verification and sequencing of pZASS-based plasmids, including pCORE
oDM0036	AACGTCTCGCTCGAATGTCTCTGTCTGGTGCTACCGG	Cloning of *bkace137* into expression vector
oDM0037	TTCGTCTCCCTCAAAGCTTAGAAAGCAACACGGTCAGCAC	Cloning of *bkace137* into expression vector
oDM0038	AACGTCTCGCTCGAATGGCTGGTCGTCTGTCTACCG	Cloning of *bkace244* into expression vector
oDM0039	TTCGTCTCCCTCAAAGCTTAGAAAGCAACAGCGTCAGCAC	Cloning of *bkace244* into expression vector
oDM0042	AACGTCTCGCTCGAATGCTGAACCGTAAAGTTATCATCTCTTG	Cloning of *bkace314* into expression vector
oDM0043	TTCGTCTCCCTCAAAGCTTAGAAAGAAACTTTGTCAGCACCTTTC	Cloning of *bkace314* into expression vector
oDM0046	AACGTCTCGCTCGAATGTCTCTGAACGGTAAAGTTATCATCAC	Cloning of *bkace15* into expression vector
oDM0047	TTCGTCTCCCTCAAAGCTTAGAAGTTAACCTGGTCACC	Cloning of *bkace15* into expression vector
oDM0091	GGTGCTGGTGGTACCCGTTTCGAATTTCTGTGCTACGACACCTCTCA CCTGTACAACC	Mutagenesis of *bkace15* E171L (GAA→CTG)
oDM0092	GGTGCTGGTGGTACCCGTTTCGAATTTGATTGCTACGACACCTCTCA CCTGTACAACC	Mutagenesis of *bkace15* E171D (GAA→GAT)
oDM0151	CGTGATGGTGACATCGTTAACTTAGGGCTGGGTTTACCCACAATGG TCGCCAA	Mutagenesis of *atoA* I25L (ATC→CTG)
oDM0152	ACAGCGCATCCAGATCTGGTGAACAGCGGCGGGCAACCGT	Mutagenesis of *atoA* A65S (GCT→AGC)

Supplementary references

1. Han, Y. *et al.* Oxaloacetate hydrolase, the C-C bond lyase of oxalate secreting fungi. *J Biol Chem* **282**, 9581–9590 (2007).
2. Chen, C., Sun, Q., Narayanan, B., Nuss, D. L. & Herzberg, O. Structure of oxalacetate acetylhydrolase, a virulence factor of the chestnut blight fungus. *J Biol Chem* **285**, 26685–26696 (2010).
3. Narayanan, B. *et al.* Structure and function of 2,3-dimethylmalate lyase, a PEP mutase/isocitrate lyase superfamily member. *J Mol Biol* **386**, 486–503 (2009).
4. Mouratou, B., Kasper, P., Gehring, H. & Christen, P. Conversion of tyrosine phenol-lyase to dicarboxylic amino acid β- lyase, an enzyme not found in nature. *Journal of Biological Chemistry* **274**, 1320–1325 (1999).
5. Bastard, K. *et al.* Revealing the hidden functional diversity of an enzyme family. *Nature Chemical Biology* **10**, 42–49 (2014).
6. Bellinzoni, M. *et al.* 3-Keto-5-aminohexanoate cleavage enzyme: a common fold for an uncommon Claisen-type condensation. *J Biol Chem* **286**, 27399–27405 (2011).
7. Noor, E. *et al.* Pathway thermodynamics highlights kinetic obstacles in central metabolism. *PLoS Comput Biol* **10**, e1003483 (2014).
8. Beber, M. E. *et al.* eQuilibrator 3.0: a database solution for thermodynamic constant estimation. *Nucleic Acids Res* **50**, D603–D609 (2022).
9. Flamholz, A., Noor, E., Bar-Even, A. & Milo, R. eQuilibrator--the biochemical thermodynamics calculator. *Nucleic Acids Res* **40**, D770-775 (2012).
10. Trudeau, D. L. *et al.* Design and *in vitro* realization of carbon-conserving photorespiration. *Proc Natl Acad Sci U S A* **115**, E11455–E11464 (2018).
11. Scheffen, M. *et al.* A new-to-nature carboxylation module to improve natural and synthetic CO2 fixation. *Nat Catal* **4**, 105–115 (2021).
12. Rohwerder, T., Rohde, M.-T., Jehmlich, N. & Purswani, J. Actinobacterial degradation of 2-hydroxyisobutyric acid proceeds via acetone and formyl-CoA by employing a thiamine-dependent lyase reaction. *Front Microbiol* **11**, 691 (2020).
13. Przybylski, D. *et al.* Exploiting mixtures of H2, CO2, and O2 for improved production of methacrylate precursor 2-hydroxyisobutyric acid by engineered *Cupriavidus necator* strains. *Appl Microbiol Biotechnol* **99**, 2131–2145 (2015).
14. Przybylski, D., Rohwerder, T., Harms, H., Yaneva, N. & Müller, R. H. Synthesis of the building block 2-hydroxyisobutyrate from fructose and butyrate by *Cupriavidus necator* H16. *Appl Microbiol Biotechnol* **97**, 8875–8885 (2013).
15. Kurteva-Yaneva, N. *et al.* Structural basis of the stereospecificity of bacterial B12-dependent 2-hydroxyisobutyryl-CoA mutase. *J Biol Chem* **290**, 9727–9737 (2015).
16. Frey, J. *et al.* Two enzymes of the acetone degradation pathway of *Desulfococcus biacutus*: coenzyme B12-dependent 2-hydroxyisobutyryl-CoA mutase and 3-hydroxybutyryl-CoA dehydrogenase. *Environmental Microbiology Reports* **10**, 283–292 (2018).
17. Weichler, M.-T. *et al.* Thermophilic coenzyme B12-dependent acyl coenzyme A (CoA) mutase from *Kyrpidia tusciae* DSM 2912 preferentially catalyzes isomerization of (*R*)-3-hydroxybutyryl-CoA and 2-hydroxyisobutyryl-CoA. *Appl Environ Microbiol* **81**, 4564–4572 (2015).

18. Rohde, M.-T., Tischer, S., Harms, H. & Rohwerder, T. Production of 2-hydroxyisobutyric acid from methanol by *Methylobacterium extorquens* AM1 expressing (*R*)-3-hydroxybutyryl coenzyme A-isomerizing enzymes. *Appl Environ Microbiol* **83**, e02622-16 (2017).

19. Chou, A., Clomburg, J. M., Qian, S. & Gonzalez, R. 2-hydroxyacyl-CoA lyase catalyzes acyloin condensation for one-carbon bioconversion. *Nat Chem Biol* **15**, 900–906 (2019).

20. Madeira, F. *et al.* Search and sequence analysis tools services from EMBL-EBI in 2022. *Nucleic Acids Res* **50**, W276–W279 (2022).

21. Waterhouse, A. *et al.* SWISS-MODEL: homology modelling of protein structures and complexes. *Nucleic Acids Res* **46**, W296–W303 (2018).

22. Walker, B. J., VanLoocke, A., Bernacchi, C. J. & Ort, D. R. The costs of photorespiration to food production now and in the future. *Annu Rev Plant Biol* **67**, 107–129 (2016).

23. Fu, X., Gregory, L. M., Weise, S. E. & Walker, B. J. Integrated flux and pool size analysis in plant central metabolism reveals unique roles of glycine and serine during photorespiration. *Nat Plants* **9**, 169–178 (2023).

24. Fernie, A. R. & Bauwe, H. Wasteful, essential, evolutionary stepping stone? The multiple personalities of the photorespiratory pathway. *Plant J* **102**, 666–677 (2020).

25. Eisenhut, M. *et al.* The photorespiratory glycolate metabolism is essential for cyanobacteria and might have been conveyed endosymbiontically to plants. *Proc Natl Acad Sci U S A* **105**, 17199–17204 (2008).

26. Nattermann, M. *et al.* Engineering a new-to-nature cascade for phosphate-dependent formate to formaldehyde conversion *in vitro* and *in vivo*. *Nat Commun* **14**, 2682 (2023).

27. Fukui, T., Ito, M. & Tomita, K. Purification and characterization of acetoacetyl-CoA synthetase from *Zoogloea ramigera* I-16-M. *Eur J Biochem* **127**, 423–428 (1982).

28. Liu, C., Wang, Q., Xian, M., Ding, Y. & Zhao, G. Dissection of malonyl-coenzyme A reductase of Chloroflexus aurantiacus results in enzyme activity improvement. *PLoS One* **8**, e75554 (2013).

29. Deatherage, D. E. & Barrick, J. E. Identification of mutations in laboratory-evolved microbes from next-generation sequencing data using breseq. *Methods Mol Biol* **1151**, 165–188 (2014).

30. Kebeish, R. *et al.* Chloroplastic photorespiratory bypass increases photosynthesis and biomass production in Arabidopsis thaliana. *Nat Biotechnol* **25**, 593–599 (2007).

31. Maier, A. *et al.* Transgenic introduction of a glycolate oxidative cycle into *A. thaliana* chloroplasts leads to growth improvement. *Front Plant Sci* **3**, 38 (2012).

32. South, P. F., Cavanagh, A. P., Liu, H. W. & Ort, D. R. Synthetic glycolate metabolism pathways stimulate crop growth and productivity in the field. *Science* **363**, eaat9077 (2019).

33. Shih, P. M., Zarzycki, J., Niyogi, K. K. & Kerfeld, C. A. Introduction of a synthetic CO_2-fixing photorespiratory bypass into a cyanobacterium. *J Biol Chem* **289**, 9493–9500 (2014).

34. Marchal, D. G. *et al.* Machine learning-supported enzyme engineering toward improved CO2-fixation of glycolyl-CoA carboxylase. *ACS Synth Biol* **12**, 3521–3530 (2023).

35. Ferguson, S. J. The ups and downs of P/O ratios: (and the question of non-integral coupling stoichiometries for oxidative phosphorylation and related processes). *Trends in Biochemical Sciences* **11**, 351–353 (1986).

36. Hinkle, P. C. P/O ratios of mitochondrial oxidative phosphorylation. *Biochimica et Biophysica Acta (BBA) - Bioenergetics* **1706**, 1–11 (2005).

37. Thoma, S. & Schobert, M. An improved *Escherichia coli* donor strain for diparental mating. *FEMS Microbiol Lett* **294**, 127–132 (2009).

38. Jensen, S. I., Lennen, R. M., Herrgård, M. J. & Nielsen, A. T. Seven gene deletions in seven days: Fast generation of *Escherichia coli* strains tolerant to acetate and osmotic stress. *Sci Rep* **5**, 17874 (2015).

39. He, H., Höper, R., Dodenhöft, M., Marlière, P. & Bar-Even, A. An optimized methanol assimilation pathway relying on promiscuous formaldehyde-condensing aldolases in *E. coli*. *Metab Eng* **60**, 1–13 (2020).

40. Kitagawa, M. *et al.* Complete set of ORF clones of *Escherichia coli* ASKA library (a complete set of *E. coli* K-12 ORF archive): unique resources for biological research. *DNA Res* **12**, 291–299 (2005).

41. Opgenorth, P. H., Korman, T. P. & Bowie, J. U. A synthetic biochemistry molecular purge valve module that maintains redox balance. *Nat Commun* **5**, 4113 (2014).

42. Zarzycki, J., Sutter, M., Cortina, N. S., Erb, T. J. & Kerfeld, C. A. *In vitro* characterization and concerted function of three core enzymes of a glycyl radical enzyme - associated bacterial microcompartment. *Sci Rep* **7**, 42757 (2017).

43. Alber, B. *et al.* Malonyl-coenzyme A reductase in the modified 3-hydroxypropionate cycle for autotrophic carbon fixation in archaeal *Metallosphaera* and *Sulfolobus* spp. *J Bacteriol* **188**, 8551–8559 (2006).

44. Wenk, S., Yishai, O., Lindner, S. N. & Bar-Even, A. An Engineering approach for rewiring microbial metabolism. *Methods Enzymol* **608**, 329–367 (2018).

45. Schulz-Mirbach, H. *et al.* On the flexibility of the cellular amination network in *E. coli*. *Elife* **11**, e77492 (2022).
