# Supplementary material for: Design and implementation of aerobic and ambient CO2-reduction as an entry-point for enhanced carbon fixation
Source: Nat Commun. 2025 Apr 1;16:3134. doi: 10.1038/s41467-025-57549-4 (PMC11961710; doi:10.1038/s41467-025-57549-4)
Supplement: Supplementary file 3 — Description of Additional Supplementary Files [file 41467_2025_57549_MOESM3_ESM.pdf]

Description of Additional Supplementary Files

File Name: Supplementary Data 1
Description: Mutations identified in evolved clones

File Name: Supplementary Data 2
Description: Plasmids

File Name: Supplementary Data 3
Description: Sequence maps of key plasmids

File Name: Supplementary Data 4
Description: Whole-genome resequencing - Changes to reference sequence

File Name: Supplementary Data 5
Description: Results of flux balance analysis (photorespiration)
