# Supplementary material for: Design and implementation of aerobic and ambient CO2-reduction as an entry-point for enhanced carbon fixation
Source: Nat Commun. 2025 Apr 1;16:3134. doi: 10.1038/s41467-025-57549-4 (PMC11961710; doi:10.1038/s41467-025-57549-4)
Supplement: Supplementary file 9 — Reporting Summary [file 41467_2025_57549_MOESM9_ESM.pdf]

nature portfolio

Corresponding author(s): Ari Satanowski, Prof. Dr. Tobias J. Erb

Last updated by author(s): Feb 16, 2025

Reporting Summary

Nature Portfolio wishes to improve the reproducibility of the work that we publish. This form provides structure for consistency and transparency in reporting. For further information on Nature Portfolio policies, see our Editorial Policies and the Editorial Policy Checklist.

Statistics

For all statistical analyses, confirm that the following items are present in the figure legend, table legend, main text, or Methods section.

n/a	Confirmed	
☐	☒	The exact sample size (*n*) for each experimental group/condition, given as a discrete number and unit of measurement
☐	☒	A statement on whether measurements were taken from distinct samples or whether the same sample was measured repeatedly
☐	☒	The statistical test(s) used AND whether they are one- or two-sided<br>*Only common tests should be described solely by name; describe more complex techniques in the Methods section.*
☒	☐	A description of all covariates tested
☒	☐	A description of any assumptions or corrections, such as tests of normality and adjustment for multiple comparisons
☐	☒	A full description of the statistical parameters including central tendency (e.g. means) or other basic estimates (e.g. regression coefficient) AND variation (e.g. standard deviation) or associated estimates of uncertainty (e.g. confidence intervals)
☒	☐	For null hypothesis testing, the test statistic (e.g. *F*, *t*, *r*) with confidence intervals, effect sizes, degrees of freedom and *P* value noted<br>*Give P values as exact values whenever suitable.*
☒	☐	For Bayesian analysis, information on the choice of priors and Markov chain Monte Carlo settings
☒	☐	For hierarchical and complex designs, identification of the appropriate level for tests and full reporting of outcomes
☒	☐	Estimates of effect sizes (e.g. Cohen's *d*, Pearson's *r*), indicating how they were calculated

Our web collection on statistics for biologists contains articles on many of the points above.

Software and code

Policy information about availability of computer code

Data collection	LC-MS data was collected using the Thermo Fisher MassHunter Workstation LC-MS Data acquisition for 6400 series Triple Quadropole Version 10.1 Build 10.1.62. Spectrophotometric data was collected using Agilent Cary WinUV Kinetics Application V.5.0.0.999. X-ray data processing was done using XDS, CCP4 v.8.0, and Phenix v1.20.
Data analysis	Custom code for flux balance analysis using the open-source package COBRApy has been deposited (DOI and repository link indicated in the manuscript "Data availability" section). Genome sequencing data was analysed using the open-source software package breseq (DOI and repository link indicated in the manuscript "Data availability" section). The corresponding raw sequencing data and reference sequences for read mapping are also provided in the Data availability section. LC-MS data was analyzed using the Thermo Fisher MassHunter Workstation Quantitative Analysis for QQQ Version 10.0 Build 10.0.707.0. Further software used were Microsoft Excel and Word, Gen5 versions 3.04 and 3.05, MatLab R2019a, GraphPad Prism 10, Geneious Prime 2023.1.2, Snapgene 6.2., PyMol Molecular Graphics System 2.5.7, Coot v.0.9.8.3, UCSF-Chimera 1.14, MUSCLE 3.8

For manuscripts utilizing custom algorithms or software that are central to the research but not yet described in published literature, software must be made available to editors and reviewers. We strongly encourage code deposition in a community repository (e.g. GitHub). See the Nature Portfolio guidelines for submitting code & software for further information.

Data

Policy information about availability of data

All manuscripts must include a data availability statement. This statement should provide the following information, where applicable:

- Accession codes, unique identifiers, or web links for publicly available datasets
- A description of any restrictions on data availability
- For clinical datasets or third party data, please ensure that the statement adheres to our policy

Data supporting the findings of this work are available within the paper and its Supplementary Information Files. Raw sequencing data from whole genome re-sequencing has been deposited on the Sequence Read Archive (accession PRJNA1124540). Links to deposited reference sequences for read mapping are provided in the data availability section. Engineered strains reported here are available for non-profit research upon request from the corresponding authors. Source data for all graphs is provided in the Source Data file.

Research involving human participants, their data, or biological material

Policy information about studies with human participants or human data. See also policy information about sex, gender (identity/presentation), and sexual orientation and race, ethnicity and racism.

Reporting on sex and gender	N/A
Reporting on race, ethnicity, or other socially relevant groupings	N/A
Population characteristics	N/A
Recruitment	N/A
Ethics oversight	N/A

Note that full information on the approval of the study protocol must also be provided in the manuscript.

Field-specific reporting

Please select the one below that is the best fit for your research. If you are not sure, read the appropriate sections before making your selection.

[x] Life sciences   [ ] Behavioural & social sciences   [ ] Ecological, evolutionary & environmental sciences

For a reference copy of the document with all sections, see nature.com/documents/nr-reporting-summary-flat.pdf

Life sciences study design

All studies must disclose on these points even when the disclosure is negative.

Sample size	Sample sizes for each experiment are specified in the figure legends. Unless otherwise specified, experiments were performed in technical triplicates.
Data exclusions	Datapoints excluded for analyses are shown in graphs and were indicated in the relevant figure legends.
Replication	Reproducibility was ensured by conducting growth experiments at least three times in culture tubes and microplate readers, with cells that were freshly prepared from glycerol stocks. At least two or three independent experimental repetitions were performed to verify our results. All attempts of replication were successful.
Randomization	Where indicated, single clones representing biological replicates were randomly chosen from culture plates.
Blinding	Blinding was not performed.

Reporting for specific materials, systems and methods

We require information from authors about some types of materials, experimental systems and methods used in many studies. Here, indicate whether each material, system or method listed is relevant to your study. If you are not sure if a list item applies to your research, read the appropriate section before selecting a response.

Materials & experimental systems

n/a	Involved in the study
☒	☐ Antibodies
☒	☐ Eukaryotic cell lines
☒	☐ Palaeontology and archaeology
☒	☐ Animals and other organisms
☒	☐ Clinical data
☒	☐ Dual use research of concern
☒	☐ Plants

Methods

n/a	Involved in the study
☒	☐ ChIP-seq
☒	☐ Flow cytometry
☒	☐ MRI-based neuroimaging

Plants

Seed stocks	N/A
Novel plant genotypes	N/A
Authentication	N/A
